# Tribbles Pseudokinase 3 Converts Sorafenib Therapy to Neutrophil‐Mediated Lung Metastasis in Hepatocellular Carcinoma

**DOI:** 10.1002/advs.202413682

**Published:** 2025-02-11

**Authors:** Xu‐Yan Wang, Yuan Liao, Rui‐Qi Wang, Yi‐Tong Lu, Ying‐Zhe Wang, Yu‐Qi Xin, Dong‐Ming Kuang, Xiang‐Ming Lao, Junying Xu, Zhi‐Ling Zhou, Kunhua Hu

**Affiliations:** ^1^ Guangdong Provincial Key Laboratory of Liver Disease Research The Third Affiliated Hospital of Sun Yat‐sen University Guangzhou 510630 China; ^2^ State Key Laboratory of Oncology in South China Guangdong Provincial Clinical Research Center for Cancer Sun Yat‐sen University Cancer Center Guangzhou 510060 China; ^3^ Guangdong Provincial Key Laboratory of Tumor Interventional Diagnosis and Treatment (No. 2021B1212040004) Zhuhai Institute of Translational Medicine Zhuhai People's Hospital (The Affiliated Hospital of Beijing Institute of Technology, Zhuhai Clinical Medical College of Jinan University) Zhuhai 519000 China; ^4^ Department of Laboratory Medicine The Third Affiliated Hospital of Sun Yat‐sen University Guangzhou 510630 China; ^5^ Department of Pharmacy Zhuhai People's Hospital (The Affiliated Hospital of Beijing Institute of Technology, Zhuhai Clinical Medical College of Jinan University) Zhuhai 519000 China; ^6^ School of Life Sciences Sun Yat‐sen University Guangzhou 510275 China; ^7^ Department of Oncology The Affiliated Wuxi People's Hospital of Nanjing Medical University Wuxi People's Hospital Wuxi Medical Center Nanjing Medical University Wuxi 214023 China

**Keywords:** hepatocellular carcinoma, lung metastasis, neutrophil, sorafenib resistance, TRIB3

## Abstract

Rapid development of resistance to sorafenib and subsequent hyperprogression in patients with advanced hepatocellular carcinoma (HCC) pose significant challenges, with the underlying mechanisms still largely unknown. Herein, sorafenib‐induced TRIB3 is identified as a liver‐specific determinant driving secondary resistance to sorafenib by facilitating the accumulation of protumorigenic neutrophils within tumors. Mechanistically, TRIB3, triggered by the sorafenib‐elicited ROS‐ER stress axis, operates in an NF‐κB‐dependent manner to upregulate CXCR1/2 ligands, subsequently promoting neutrophil recruitment into tumors. These enriched neutrophils enhance epithelial‐mesenchymal transition processes in malignant cells through the oncostatin M‐STAT3 pathway, thereby repurposing the therapeutic efficacy of sorafenib away from anti‐angiogenesis and toward lung metastasis. Clinically, elevated TRIB3 expression indicates inferior survival and unfavorable clinical efficacy of sorafenib in HCC patients. Correspondingly, strategies that either inhibiting TRIB3 upregulation or blocking its downstream signaling successfully augment the therapeutic efficacy of sorafenib and prevent sorafenib‐induced hyperprogression in vivo. The study thus identifies a pivotal mechanism of sorafenib resistance in HCC, centered on the TRIB3‐mediated recruitment of protumorigenic neutrophils and subsequent disease hyperprogression.

## Graphical abstract

Rapid resistance to sorafenib and subsequent hyperprogression pose challenges to HCC treatment. This study identifies TRIB3 as determinant driving secondary resistance to sorafenib. Mechanistically, TRIB3, triggered by sorafenib‐elicited ROS‐ER stress, operates in an NF‐κB‐dependent manner to upregulate CXCR1/2 ligands and recruit neutrophils. It suggests a novel TRIB3‐based combination therapapeutic strategy.

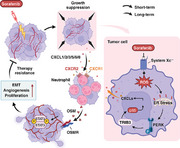

## 1 Introduction

Hepatocellular carcinoma (HCC) typically arises from inflamed liver tissues, with its progression hinging on the interplay between endogenous oncogenes within the tumor cells and the surrounding microenvironment.^[^ ^1^, ^2^, ^3^, ^4^, ^5^ ^]^ Notably, most HCC patients receive their diagnosis at an advanced stage, rendering surgical intervention impractical and necessitating reliance on comprehensive treatment approaches, such as chemotherapy, targeted therapy, and immunotherapy.^[^ ^6^, ^7^ ^]^ However, how these treatments alter the genetic characteristics of tumor cells and the microenvironment remains elusive. Unraveling these effects is essential not only for identifying markers to access therapeutic efficacy but also for developing innovative therapeutic strategies.

Sorafenib, by inhibiting multiple kinase activities and pathways, including vascular endothelial growth factor receptor, platelet‐derived growth factor receptor, and Ras/Raf/MEK/ERK signaling, targets both cancer cells and the angiogenic microenvironment.^[^ ^8^, ^9^ ^]^ Currently, sorafenib serves as the first‐line agent for patients with advanced HCC and has shown significant benefits.^[^ ^10^, ^11^, ^12^ ^]^ However, most patients develop drug resistance within a short time period, leading to subsequent hyperprogressive disease, with its mechanism unknown.^[^ ^10^, ^13^, ^14^ ^]^ Intriguingly, HCC patients tend to develop resistance to sorafenib more swiftly than patients with other tumors, indicating the presence of HCC‐specific resistance mechanisms.^[^ ^12^, ^15^, ^16^, ^17^ ^]^ Elucidating the underlying mechanisms of negative feedback induced by sorafenib in HCC is crucial for devising strategies to extend the effectiveness of sorafenib.

The mammalian Tribbles (TRIB) family, comprising TRIB1, TRIB2, and TRIB3, is classified as pseudokinases that function as adaptor proteins to modulate and integrate various signaling pathways.^[^ ^18^, ^19^, ^20^ ^]^ TRIB3, distinct from its counterparts, is highly expressed in the liver.^[^ ^21^ ^]^ Recent studies have shown that TRIB3 can lead to the formation of cold tumors, suggesting a role in remodeling the immune microenvironment.^[^ ^22^ ^]^ Additionally, TRIB3 functions as a stress sensor in response to diverse stressors and has been identified as an oncoprotein in both solid tumors and hematological cancers.^[^ ^23^, ^24^, ^25^, ^26^ ^]^ Herein, we identified sorafenib‐induced TRIB3 as a pivotal determinant driving acquired resistance to sorafenib by facilitating the accumulation of protumorigenic neutrophils within tumors. Mechanistically, TRIB3 triggered by sorafenib‐elicited ROS‐ER stress axis operated in an NF‐κB‐dependent manner to upregulate CXCR1/2 ligands, subsequently promoting neutrophil recruitment into tumors. These enriched neutrophils enhanced epithelial‐mesenchymal transition (EMT) processes in malignant cells through the oncostatin M (OSM)‐STAT3 pathway, thereby repurposing the therapeutic efficacy of sorafenib away from anti‐angiogenesis and toward lung metastasis. Clinically, elevated TRIB3 expression indicates inferior survival and unfavorable clinical efficacy of sorafenib in HCC patients. Correspondingly, strategies that abrogate such negative feedback regulation by either inhibiting TRIB3 upregulation or blocking its downstream signaling successfully augment therapeutic efficacy of sorafenib and prevent sorafenib‐induced hyperprogression in vivo.

## 2 Results

### 2.1 Inducing TRIB3 by Sorafenib Promotes Therapeutic Resistance and Metastasis

**Figure 1:**
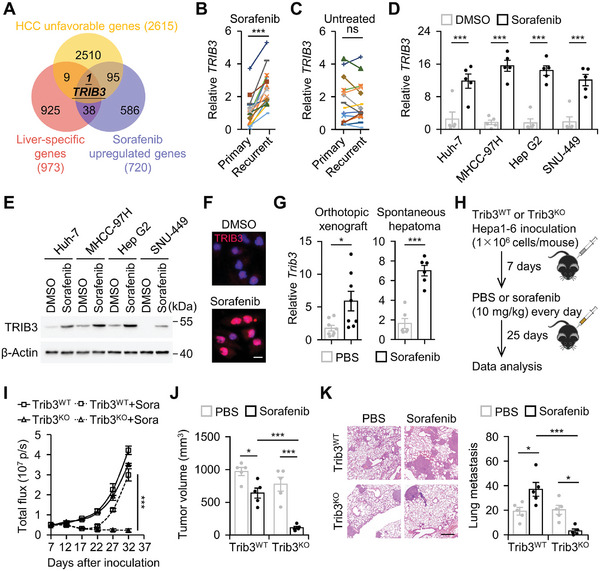
Inducing TRIB3 by sorafenib promotes therapeutic resistance and metastasis. A) Venn diagram showing the overlap between liver‐specific genes (The Human Tissue Specific Proteome section of the Human Protein Atlas), genes associated with an unfavorable prognosis in HCC (The Liver Cancer Proteome section of the Human Protein Atlas), and genes significantly upregulated by sorafenib treatment in HCC (GEO: GSE186280). B,C) Comparison of TRIB3 levels between recurrent tumor tissue and matched primary resected sample from HCC patients who received sorafenib therapy before secondary resection (B, *n* = 15) or those who did not receive additional treatment before secondary resection (C, *n* = 15). D,E) Human hepatoma cells were treated with DMSO or sorafenib for 48 h (each *n* = 5). The expression of TRIB3 was determined by real‐time PCR (D) and immunoblotting (E). F) Huh‐7 cells were treated with DMSO or sorafenib for 48 h. The levels of TRIB3 expression were analyzed by immunofluorescence (*n* = 3). Scale bar, 50 µm. G) C57BL/6 mice, either bearing orthotopic xenografts of Hepa1‐6 cells or with spontaneous hepatomas, were treated with sorafenib or PBS as described in Figure S1C (Supporting Information) (each *n* = 8 for orthotopic xenograft; each *n* = 6 for spontaneous hepatoma). Trib3 expression in hepatoma was determined by real‐time PCR. H–K) Wild‐type or Trib3‐deficient Hepa1‐6 hepatoma‐bearing mice were injected with PBS or sorafenib as described (H) (each *n* = 5). Tumor growth monitored by bioluminescence imaging (I), tumor volume (J), and lung metastasis (K) of hepatoma were analyzed. Scale bar, 625 µm (K). Data represent mean ± SEM of three independent experiments. ^*^ *p* < 0.05, ^***^ *p* < 0.001, Student's *t* test (B–D, and G), two‐way ANOVA with Tukey's post test (I–K).

To identify the genetic determinants driving secondary resistance in hepatocellular carcinoma (HCC) patients treated with sorafenib, we analyzed liver‐specific genes exhibiting upregulation subsequent to sorafenib administration, indicative of poor prognosis in HCC. Surprisingly, among these genes, TRIB3 emerged as the singular liver‐specific gene signaling adverse prognosis and displaying sorafenib‐induced upregulation in HCC (**Figure** **1**A; Figure S1A,B, Supporting Information). Furthermore, we assessed TRIB3 expression in recurrent HCC patients who had undergone sorafenib therapy prior to secondary resection, revealing a marked elevation of TRIB3 in recurrent tumor tissues compared to matched primary resected samples (Figure 1B, *n* = 15, *p* < 0.001). Conversely, TRIB3 expression exhibited marginal alterations in recurrent HCC tissues from patients not receiving additional treatment before secondary resection (Figure 1C, *n* = 15). To test whether induction of TRIB3 by sorafenib is dependent on in vivo cancer environments, we directly subjected hepatoma cell lines—Huh‐7, MHCC‐97H, Hep G2, and SNU‐449—to sorafenib in vitro. Notably, sorafenib alone, devoid of in vivo cancer environments, elicited a minimum threefold increase in TRIB3 expression, with a predominant localization of TRIB3 within the nucleus (Figure 1D–F). Thus, TRIB3 emerges as a potent prognostic marker, directly upregulated by sorafenib treatment.

To ascertain whether the upregulation of TRIB3 induced by sorafenib contributes to therapeutic resistance, we established sorafenib therapeutic strategy in mice bearing orthotropic Hepa1‐6 cell‐derived hepatoma or hydrodynamic force‐induced spontaneous hepatoma. As anticipated, sorafenib administration robustly elevated TRIB3 expression in both in vivo models (Figure 1G; Figure S1C,D, Supporting Information). Examination of hepatoma growth kinetics revealed a paradoxical response to sorafenib, characterized by initial treatment phases inducing efficient growth suppression, succeeded by a rebound in growth, consistent with the clinical efficacy profile of sorafenib in treating HCC patients (Figure 1H,I). Notably, also in such a model, knockout of TRIB3 accentuated sorafenib's therapeutic effect, fostering sustained hepatoma regression, although this treatment marginally affected hepatoma growth in the absence of sorafenib (Figure 1H–J; Figure S1E,F, Supporting Information). More interestingly, prolonged sorafenib exposure resulted in increased lung metastasis of hepatoma, an effect nullified upon TRIB3 knockout (Figure 1K). Consistent with these observations, elevated TRIB3 expression in human HCC correlated positively with increased intrahepatic metastasis and vascular invasion (Figure S1G,H, Supporting Information). In addition, HCC patients exhibiting elevated tumor expression of TRIB3 frequently displayed poor responsiveness to sorafenib therapy (Figure S1I, Supporting Information). In aggregate, our findings suggest that sorafenib‐induced TRIB3 upregulation may precipitate a therapeutic transition toward lung metastasis in HCC.

### 2.2 TRIB3 is Triggered by Sorafenib‐Elicited ROS‐ER Stress Axis in HCC

**Figure 2:**
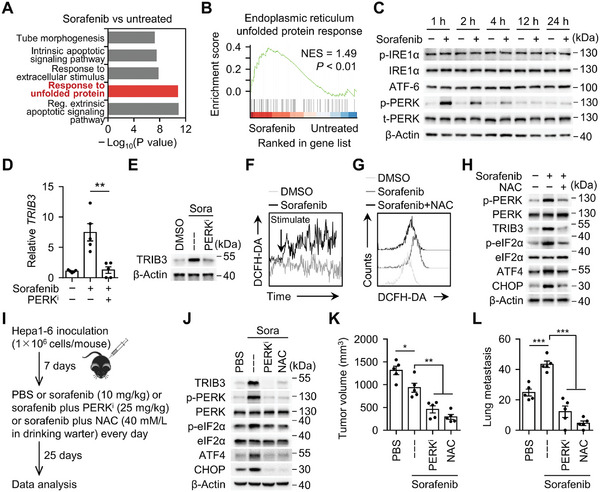
TRIB3 is triggered by sorafenib‐elicited ROS‐ER stress axis in HCC. A) Functional annotation of significantly upregulated genes in sorafenib‐treated Hep G2 cells (GSE186280) were analyzed by Metascape. The top 5 enrichment GO terms are listed. B) Gene Set Enrichment Analysis (GSEA) of endoplasmic reticulum unfolded protein response in sorafenib‐treated versus untreated Hep G2 cells (GSE186280). C) Huh‐7 cells were treated with DMSO or sorafenib for indicated time. Activation of indicated pathways was analyzed by immunoblotting (*n* = 3). D,E) Huh‐7 cells were treated with DMSO or sorafenib in the absence (—) or presence of PERK inhibitor (PERK^i^) for 48 h (*n* = 5), the expression of TRIB3 were determined by real‐time PCR (D) and immunoblotting (E), respectively. F) Dynamic assessment of reactive oxygen species (ROS) generation induced by sorafenib. Huh‐7 cells were stained with the fluorescent ROS probe DCFH‐DA, and were subsequently treated with sorafenib (10 µm). Thereafter, the DCF fluorescence intensity in these cells was analyzed by flow cytometry (*n* = 3). G,H) Huh‐7 cells were treated with DMSO or sorafenib in the absence or presence of the ROS scavenger NAC (*n* = 3). Thereafter, the DCF fluorescence intensity was analyzed by flow cytometry (48 h, G). PERK activation (1 h) and its downstream effector molecules, TRIB3, eIF2α, ATF4, and CHOP (48 h) were determined by immunoblotting (H). I–L) Hepa1‐6 hepatoma‐bearing mice were left untreated or treated with PERK inhibitor or NAC in the presence of sorafenib for 25 days as described in (I) (each *n* = 5). Protein levels of TRIB3 and PERK pathway (J), tumor volume (K) and lung metastasis (L) of hepatoma were analyzed. Data represent mean ± SEM of three independent experiments (D, K, and L). ^**^ *p* < 0.01, ^***^ *p* < 0.001, one‐way ANOVA with Tukey's post test (D, K, and L).

We proceeded to investigate the potential mechanisms underlying TRIB3 induction upon sorafenib therapy in HCC. Through transcriptomic analysis, we identified 556 genes exhibiting at least a twofold upregulation in hepatoma cells following sorafenib treatment and annotated these genes using Gene Ontology (GO) (**Figure** **2**A). Pathway related to the unfolded protein response (UPR) was intensively enriched in hepatoma cells treated with sorafenib, which was further confirmed by Gene Set Enrichment Analysis (GSEA), implicating a potential involvement of endoplasmic reticulum (ER) stress in sorafenib‐mediated TRIB3 induction (Figure 2A,B). Notably, ER stress can activate downstream effectors such as ATF6, IRE1, or PERK to orchestrate the UPR, exerting both physiological and pathological effects.^[^ ^27^ ^]^ Indeed, only the PERK signaling pathway, and not ATF6 or IRE1 signaling, was detected in hepatoma cells treated with sorafenib (Figure 2C). Furthermore, the eIF2α/ATF4/CHOP axis, a crucial downstream cascade of PERK signaling, exhibited marked activation in these cells (Figure S2A, Supporting Information). In support, inhibition of the PERK/eIF2α/ATF4/CHOP pathway effectively attenuated sorafenib‐induced TRIB3 upregulation, aligning with previous reports implicating transcription factors ATF4 and CHOP cooperating in the modulation of TRIB3 expression (Figure 2D,E; Figure S2B–E, Supporting Information).^[^ ^28^ ^]^ Thus, sorafenib operates via a PERK‐related ER stress pathway to trigger TRIB3 in HCC.

Sorafenib has been reported to inhibit system xc(‐), a pivotal component of cellular antioxidant systems.^[^ ^29^, ^30^ ^]^ Consequently, we sought to investigate whether such a mechanism is also responsible for PERK‐mediated TRIB3 upregulation in hepatoma cells following sorafenib treatment. Consistently, sorafenib treatment did lead to the accumulation of reactive oxygen species (ROS) in hepatoma cells, and using N‐acetylcysteine (NAC) to clear ROS markedly suppressed the activation of the PERK/eIF2α/ATF4/CHOP signaling pathway, as well as the upregulation of TRIB3, induced by sorafenib (Figure 2F–H). Furthermore, in support of the findings demonstrating TRIB3's promotion of hepatoma growth and lung metastasis, treatment with a PERK inhibitor or NAC also inhibited hepatoma growth and lung metastasis in mice (Figure 2I–L). These results underscore the role of ROS‐mediated PERK activation in TRIB3‐induced hepatoma progression.

### 2.3 In Vivo Milieus Dictate TRIB3's Protumorigenic Properties

**Figure 3:**
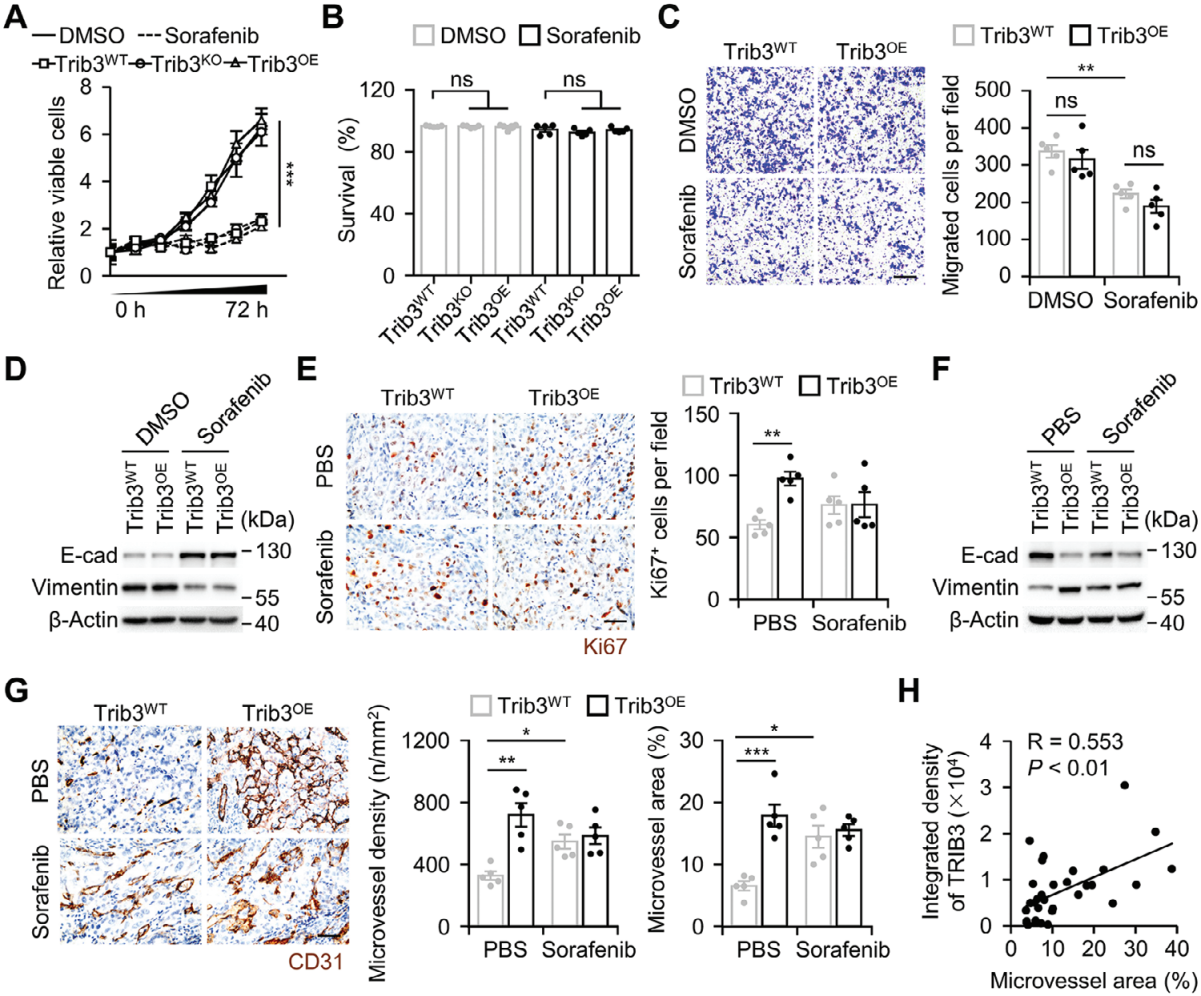
In vivo milieus dictate TRIB3's protumorigenic properties. A–C) Trib3^WT^, Trib3^KO^, or Trib3^OE^ Hepa1‐6 cells were treated with DMSO or sorafenib. Cell proliferation was measured by CCK8 assay (A). Cell apoptosis (48 h) was measured by flow cytometry (B). Migration of the cells (C, 48 h) was determined (*n* = 5). Scale bar, 200 µm. D) Trib3^WT^ or Trib3^OE^ Hepa1‐6 cells were treated with DMSO or sorafenib. Proteins of EMT genes were determined by immunoblotting (*n* = 3). E–G) Trib3^WT^ or Trib3^OE^ Hepa1‐6 hepatoma‐bearing mice were injected with PBS or sorafenib as described in Figure S3B (Supporting Information) (each *n* = 5). After 25 days of treatment, the proliferation of hepatoma cells (E, scale bar, 50 µm), morphology and area of microvessels in tumor (G, scale bar, 50 µm) were analyzed by immunohistochemistry using αKi67 and αCD31 antibody, respectively. Proteins of EMT genes in mouse tumor tissues were determined by immunoblotting (F). H) Correlation between TRIB3 expression and microvessel area in tumors from 32 HCC patients were analyzed by immunohistochemistry using αTRIB3 and αCD34 antibody. Integrated TRIB3 density and microvessel area was determined by IMMAGE J software. Data represent mean ± SEM of three independent experiments (A–C, E, and G). ^*^ *p* < 0.05, ^**^ *p* < 0.01, ^***^ *p* < 0.001, two‐way ANOVA with Tukey's post test (A, C, E, and G), one‐way ANOVA with Tukey's post test (B). P value and R value were calculated based on the analysis of Pearson's correlation (H).

After elucidating the mechanism underlying sorafenib‐induced TRIB3 upregulation in HCC, we investigated the protumorigenic properties of TRIB3. Remarkably, both overexpression and knockout of TRIB3 in an in vitro culture system failed to significantly impact the proliferation or survival of hepatoma cells in the presence or absence of sorafenib, despite sorafenib's inherent ability to inhibit cell proliferation, aligning with established knowledge (**Figure** **3**A,B). Subsequently, given the successful suppression of sorafenib‐induced lung metastasis in vivo upon TRIB3 knockout, we explored whether TRIB3 signaling initiated epithelial‐mesenchymal transition (EMT) in hepatoma cells. However, regardless of whether treated with sorafenib or not, overexpression of TRIB3 did not affect the migration of liver cancer cells, nor did it alter the expression of E‐cadherin and vimentin (Figure 3C,D). Similarly, TRIB3 knockout minimally impacted the EMT process in hepatoma cells (Figure S3A, Supporting Information). These findings collectively suggest that in the absence of an in vivo milieu, TRIB3 loses its capacity to drive hepatoma growth and metastasis.

We subsequently examined the impact of TRIB3 on cancer hallmarks in hepatoma‐bearing mice. In contrast to in vitro observations, in vivo overexpression of TRIB3 significantly enhanced hepatoma cell proliferation and EMT, as evidenced by increased Ki67 expression in hepatoma cells and alterations in vimentin and E‐cadherin levels under long‐term sorafenib treatment (Figure 3E,F; Figure S3B, Supporting Information). Additionally, analysis of tumor vasculature density and area demonstrated that TRIB3 overexpression promoted angiogenesis, resulting in increased sinusoidal vasculature within tumors (Figure 3G). Consistent with these findings, elevated TRIB3 expression in human HCC samples positively correlated with an increased area of tumor vasculature (Figure 3H). Notably, TRIB3 overexpression did not alter the expression profile of angiogenesis‐related genes in vitro (Figure S3C, Supporting Information), suggesting that TRIB3 may exert its effects indirectly, potentially through modulation of the tumor microenvironments, to promote tumor growth, angiogenesis, and metastasis.

### 2.4 Neutrophils are Essential for TRIB3‐Driven Pro‐Tumorigenesis

**Figure 4:**
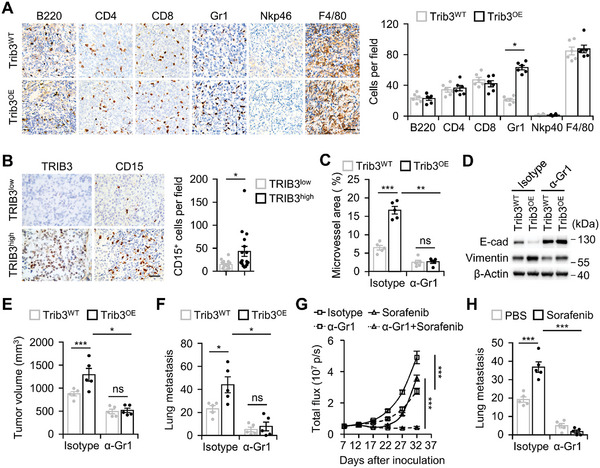
Neutrophils are essential for TRIB3‐driven pro‐tumorigenesis. A) Effects of Trib3 overexpression on the infiltration of B cell (B220^+^), T helper cell (CD4^+^), cytotoxic T cell (CD8^+^), neutrophil (Gr1^+^), NK cell (NKp46^+^), and macrophage (F4/80^+^) in mouse hepatoma (*n* = 6, scale bar, 50 µm). B) Association of TRIB3 expression and infiltration of CD15^+^ neutrophils in tumor tissues from HCC patients (*n* = 32). Scale bar, 50 µm. C–F) Trib3^WT^ or Trib3^OE^ Hepa1‐6 hepatoma‐bearing mice were treated with αGr‐1 or isotype antibody as described in Figure S4D (Supporting Information) (each *n* = 5). Angiogenesis (C), proteins of EMT genes (D), tumor volume (E), and lung metastasis (F) of hepatoma were analyzed. G,H) Hepa1‐6 hepatoma‐bearing mice were injected with sorafenib or PBS in the presence or absence of αGr‐1 antibody as described in Figure S4F (Supporting Information) (each *n* = 5). Tumor growth monitored by bioluminescence imaging (G) and lung metastasis of hepatoma were analyzed (H). Data represent mean ± SEM of three independent experiments (A–C, and E–H). ^*^ *p* < 0.05, ^***^ *p* < 0.001, Student's *t* test (A,B), two‐way ANOVA with Tukey's post test (C, and E–H).

We now asked how elevated TRIB3 levels influence the microenvironment of HCC. Utilizing RNA sequencing, we identified 97 genes exhibiting upregulation in TRIB3‐overexpressing hepatoma tissue and annotated these genes using GO. Among the top 10 enriched GO terms, pathways related to neutrophil migration, activation, and degranulation were intensively enriched. Additionally, pathways involving pathological processes, including cellular stress response, NF‐κB signaling, cell survival, metabolism, and angiogenesis, were also observed (Figure S4A,B, Supporting Information). Thus, elevated TRIB3 may promote hepatoma progression through a pathway dependent on neutrophil activity. Consistent with this, a selective increase in Gr1^+^ neutrophils was detected in TRIB3‐overexpressing mouse hepatoma (**Figure** **4**A). In contrast, the infiltration of other cell types, including B220^+^ cells, CD4^+^ cells, CD8^+^ cells, NKp46^+^ cells, and F4/80^+^ cells, showed marginal changes in that tissue (Figure 4A). Furthermore, elevated TRIB3 expression in human HCC samples positively correlated with increased neutrophil infiltration, underscoring the essential role of neutrophils in mediating TRIB3's protumorigenic properties (Figure 4B). Indeed, during sorafenib treatment, both TRIB3 expression and neutrophil infiltration were progressively upregulated in the Hepa1‐6 hepatoma (Figures S1D and S4C, Supporting Information).

Subsequently, we employed an anti‐Gr1 antibody to specifically deplete neutrophils in mice bearing wildtype or TRIB3‐overexpressing hepatoma (Figure S4D,E, Supporting Information). Consistent with our hypothesis, neutrophil depletion effectively attenuated the aggressive angiogenesis and the enhanced EMT process in hepatoma driven by TRIB3 overexpression (Figure 4C,D). Accordingly, in the neutrophil‐depleted mouse model, TRIB3 overexpression failed to promote hepatoma growth and lung metastasis (Figure 4E,F). Moreover, notably in the neutrophil‐depleted mouse model, sorafenib treatment resulted in sustained hepatoma regression and did not exacerbate the metastatic potential of hepatoma (Figure 4G,H; Figure S4F, Supporting Information), and this effect was comparable to that observed in TRIB3 knockout mice. Thus, neutrophil depletion within tumors can circumvent TRIB3‐induced therapeutic resistance to sorafenib.

### 2.5 Elevated TRIB3 Attracts Neutrophils Through Boosting NF‐κB‐CXCL Signaling

**Figure 5:**
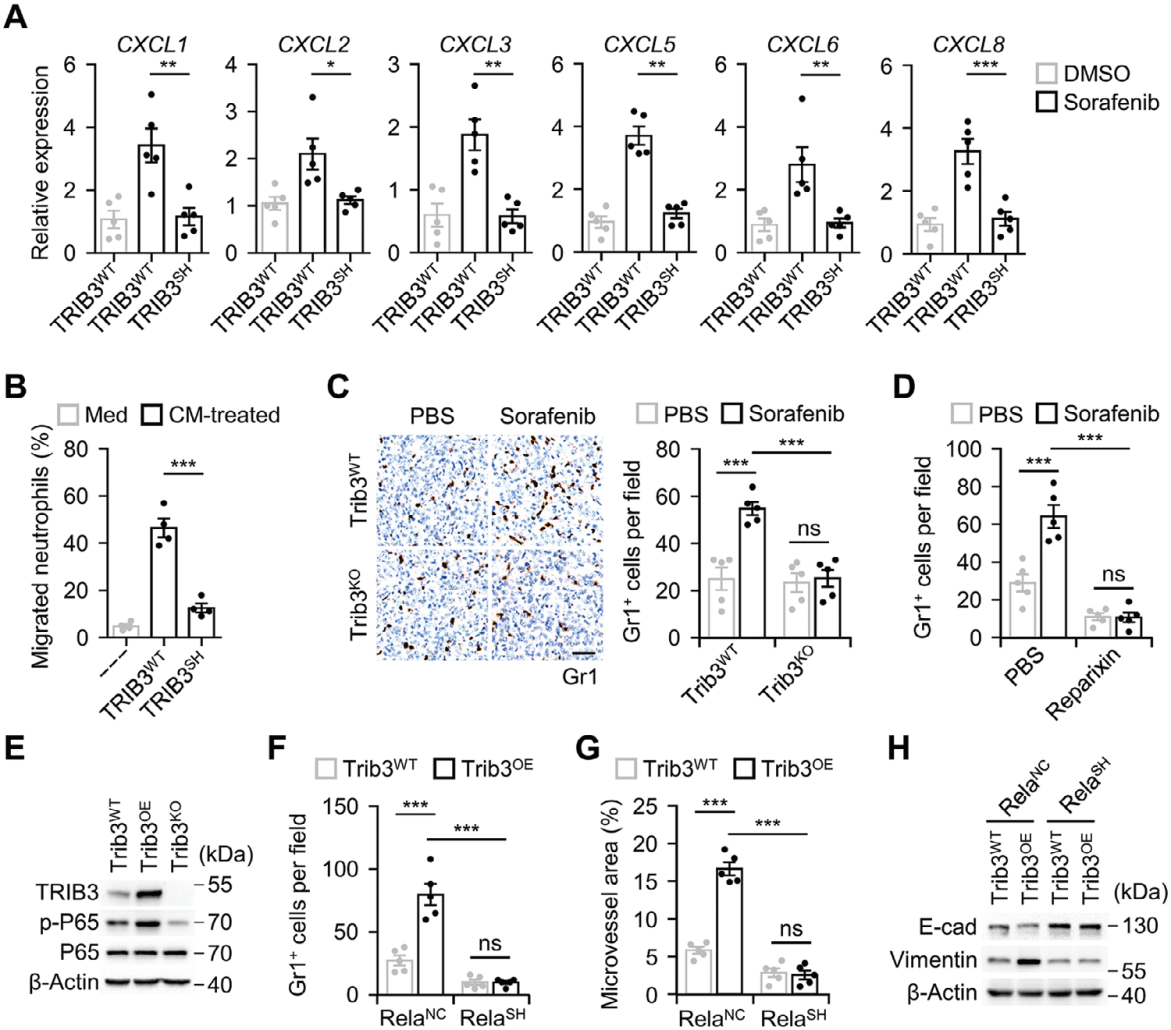
Elevated TRIB3 attracts neutrophils through boosting NF‐κB‐CXCL signaling. A) Expression of CXCR1/2 ligands in wild‐type Huh‐7 cells (TRIB3^WT^) or TRIB3‐knockdown Huh‐7 cells (TRIB3^SH^) treated with DMSO or sorafenib for 48 h (*n* = 5). B) Neutrophils were left untreated (Med) or were treated with a conditioned medium (CM‐treated) from sorafenib‐treated wild‐type (TRIB3^WT^) or TRIB3‐knockdown (TRIB3^SH^) Huh‐7 cells. Migration of neutrophils was determined (*n* = 4). C) Wild‐type or Trib3‐deficient Hepa1‐6 hepatoma‐bearing mice were injected with PBS or sorafenib as described in (Figure 1H) (each *n* = 5). Infiltration of Gr1^+^ neutrophils in tumors was analyzed by immunohistochemistry. Scale bar, 50 µm. D) Hepa1‐6 hepatoma‐bearing mice were injected with PBS or sorafenib in the absence or presence of CXCR1/2 inhibitor (Reparixin) as described in Figure S5D (Supporting Information) (each *n* = 5). Infiltration of Gr1^+^ neutrophils in tumors was analyzed. E) Protein levels of TRIB3 and activation of NF‐κB pathway in sorafenib‐treated Trib3^WT^, Trib3^OE^, or Trib3^KO^ Hepa1‐6 cells were determined (*n* = 3). F–H) Wild‐type (Trib3^WT^ or Rela^NC^), or Trib3‐overexpressing (Trib3^OE^), or p65‐knockdown (Rela^SH^), or p65‐knockdown plus Trib3‐overexpressing Hepa1‐6 cells were inoculated in liver of C57BL/6 mice as described in Figure S5H (Supporting Information) (each *n* = 5). Infiltration of Gr1^+^ neutrophils (F), area of microvessels (G), and proteins of EMT genes (H) were determined. Data represent mean ± SEM of three independent experiments (A–D, F, and G). ^*^ *p* < 0.05, ^**^ *p *< 0.01, ^***^ *p* < 0.001, one‐way ANOVA with Tukey's post test (A, and B), two‐way ANOVA with Tukey's post test (C, D, F, and G).

Having established the pivotal role of neutrophils in TRIB3‐mediated resistance to sorafenib, we delved into the mechanisms underlying the heightened accumulation of neutrophils in tumors in response to elevated TRIB3 levels. Notably, the chemokine receptors CXCR1/2 play a central role in facilitating neutrophil migration from the peripheral blood to local tissues.^[^ ^31^, ^32^, ^33^ ^]^ Intriguingly, leveraging single‐cell sequencing data from human HCC tumors, we discovered that hepatoma cells expressing TRIB3 were the predominant source of CXCR1/2 ligands, including CXCL1, CXCL2, CXCL3, CXCL5, CXCL6, and CXCL8 (Figure S5A, Supporting Information). Also, compared to their wildtype counterparts, hepatoma cells overexpressing TRIB3 exhibited significantly elevated levels of CXCR1/2 ligands (Figure S5B,C, Supporting Information). Similarly, sorafenib that induced TRIB3 upregulation also enhanced the expression of CXCR1/2 ligands, consequently augmenting neutrophil recruitment in vitro, a phenomenon that was effectively mitigated upon TRIB3 knockdown using shRNA (**Figure** **5**A,B). Thus, the elevation of TRIB3 levels by sorafenib appears to facilitate neutrophil recruitment by promoting the expression of CXCR1/2 ligands. Consistent with this, sorafenib treatment led to increased infiltration of Gr1^+^ neutrophils in hepatoma tissues, an effect that was absent in TRIB3 knockout hepatoma cells or was suppressed by administration of the CXCR1/2 inhibitor Reparixin (Figure 5C,D; Figure S5D, Supporting Information).

To dissect the mechanisms underlying TRIB3‐mediated upregulation of CXCR1/2 ligands, we scrutinized RNA‐sequencing data of HCC tumors obtained from the Cancer Genome Atlas. Our analysis revealed a positive correlation between increased NF‐κB signature scores and elevated expression levels of CXCR1/2 ligands (Figure S5E, Supporting Information). Moreover, activation of NF‐κB signaling was observed in hepatoma cells upon sorafenib treatment, despite the suppression of several other signaling pathways during this process (Figure S5F, Supporting Information). Similarly, in sorafenib‐treated hepatoma cells, overexpression of TRIB3 amplified NF‐κB signaling activation, while its knockout attenuated this effect (Figure 5E). These findings suggest that NF‐κB signaling may serve as a bridge linking elevated TRIB3 levels to the upregulation of CXCR1/2 ligands in hepatoma cells treated with sorafenib. Indeed, inhibiting the NF‐κB pathway abolished TRIB3‐induced upregulation of CXCR1/2 ligands (Figure S5G, Supporting Information). Accordingly, inhibition of NF‐κB signaling activity through p65 knockdown effectively countered TRIB3‐mediated neutrophil infiltration, thereby mitigating subsequent enhancements in angiogenesis and EMT within hepatoma tissue (Figure 5F–H; Figure S5H, Supporting Information).

### 2.6 Neutrophils Suppress Therapeutic Efficacy of Sorafenib via OSM/STAT3 Axis

**Figure 6:**
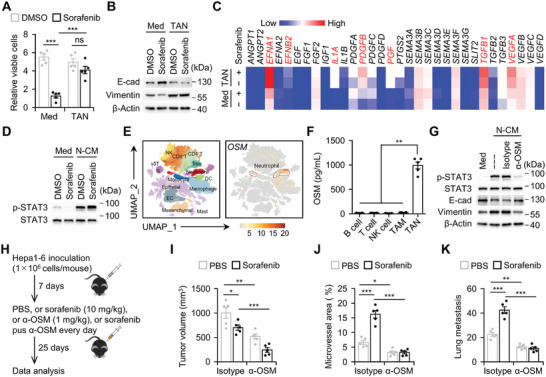
Neutrophils suppress therapeutic efficacy of sorafenib via OSM/STAT3 axis. A–C) Huh‐7 cells were treated with DMSO or sorafenib in the absence or presence of tumor‐associated neutrophils (TAN) in the upper chamber of a transwell plate (*n* = 5). Cell proliferation (96 h, A), proteins of EMT genes (48 h, B), and expression of angiogenesis‐related genes (4 h, C) were determined. D) Huh‐7 cells were treated with DMSO or sorafenib in the absence or presence of conditioned medium from tumor‐associated neutrophils (N‐CM) for 2 h. Activation of STAT3 pathway was determined by immunoblotting (*n* = 3). E) Uniform Manifold Approximation and Projection (UMAP) plots showing expression of OSM indifferent cell types in HCC tumors. Each cluster is color‐coded according to cell type. Cluster annotations are indicated in the Figure (http://meta‐cancer.cn:3838/scPLC/). F) OSM concentration in culture supernatant of indicated immune cells isolated from HCC tumors was determined by enzyme‐linked immunosorbent assay (ELISA) (*n* = 5). G) Huh‐7 cells were left untreated or pre‐treated with isotype or OSM neutralizing antibody (α‐OSM, 3 µg mL^−1^) in the presence of N‐CM. Activation of STAT3 pathway (2 h) and proteins of EMT genes (48 h) were determined by immunoblotting (*n* = 3). H–K) Hepa1‐6 hepatoma‐bearing mice were injected with PBS or sorafenib in the absence or presence of OSM neutralizing antibody (α‐OSM) as described in (H) (each *n* = 5). Tumor volume (I), area of microvessels (J), and lung metastasis (K) of hepatoma were analyzed. Data represent mean ± SEM of three independent experiments (A, F, and I–K). ^*^ *p* < 0.05, ^***^ *p* < 0.001, two‐way ANOVA with Tukey's post test (A, and I–K), one‐way ANOVA with Tukey's post test (F).

The results presented above indicate that sorafenib exerts its therapeutic effects by inhibiting the proliferation, migration, and proangiogenic activity of hepatoma cells. Indeed, in the presence of neutrophils in the upper chamber of a transwell plate, sorafenib was unable to inhibit the proliferation, EMT, or pro‐angiogenic activity of hepatoma cells, suggesting that neutrophils may counteract the therapeutic effects of sorafenib through a paracrine mechanism (**Figure** **6**A–C).

Given sorafenib's role as a multikinase inhibitor, capable of attenuating RAF/MEK/ERK (MAPK) and AKT signaling pathways, we investigated whether soluble factors released by neutrophils could hinder sorafenib's therapeutic effects by restoring those signaling pathway activities. However, exposure of hepatoma cells to conditioned medium from neutrophil cultures (N‐CM) minimally impacted the phosphorylation of MAPK and AKT pathways, which were suppressed by sorafenib treatment, despite the N‐CM restoring malignant features of those cells (Figure 6A–C; Figure S6A, Supporting Information). These findings suggest that neutrophils may activate alternative signaling mechanisms to counteract sorafenib's therapeutic efficacy. Notably, elevated neutrophils were observed in human HCC samples, positively correlating with angiogenesis progression and intrahepatic metastasis (Figure S6B–D, Supporting Information). Additionally, STAT3 signaling, crucial for tumor malignancy,^[^ ^34^, ^35^, ^36^ ^]^ was markedly activated in hepatoma cells exposed to N‐CM, minimally impacted by sorafenib treatment (Figure 6D). Accordingly, STAT3 inhibition in hepatoma cells mitigated the neutrophil‐conditioned medium's suppressive effects on sorafenib efficacy, highlighting a STAT3‐dependent mechanism through which neutrophil‐released soluble factors inhibit sorafenib's therapeutic efficacy (Figure S6E–G, Supporting Information).

We conducted further investigation into the factors utilized by neutrophils to impede the therapeutic effectiveness of sorafenib. Leveraging single‐cell sequencing data, we identified oncostatin M (OSM) as a critical STAT3‐associated factor exclusively expressed by neutrophils within HCC tumors (Figure 6E,F). Subsequent experiments demonstrated that neutralizing OSM activity with a specific antibody markedly suppressed N‐CM‐induced STAT3 activation and inhibited the EMT process in hepatoma cells (Figure 6G). Notably, in hepatoma‐bearing mice, in vivo neutralization of OSM activity significantly augmented the therapeutic efficacy of sorafenib, resulting in more effective suppression of hepatoma growth, angiogenesis, and lung metastasis (Figure 6H–K). Collectively, these findings underscore the essential role of neutrophil‐derived OSM in mediating the suppression of sorafenib's therapeutic efficacy in HCC tumors.

## 3 Discussion

HCC demonstrates a pronounced tendency to develop resistance to sorafenib‐targeted therapy. This study uncovered a novel mechanism underlying the developed resistance to sorafenib in hepatocellular carcinoma (HCC), highlighting the pivotal role of TRIB3 in this process. By employing multiple complementary approaches, we delineated the induction, biologic function, and clinical relevance of TRIB3 in the tumor microenvironment of patients with HCC.

HCC, characterized by its immune‐suppressive milieu and chronic viral infections, employs unique protumorigenic mechanisms distinct from other tumors.^[^ ^37^, ^38^, ^39^, ^40^, ^41^ ^]^ Particularly, when receive sorafenib, the cornerstone systemic therapy in the first‐line treatment, HCC patients develop resistance more rapidly than other tumors, underscoring the necessity of identifying liver cancer‐specific resistance mechanisms.^[^ ^12^, ^15^, ^16^, ^17^ ^]^ Our investigation identified TRIB3 as a crucial determinant of sorafenib resistance. Unlike other pseudokinases, TRIB3 is selectively expressed in liver and modulates diverse signaling pathways.^[^ ^19^, ^21^ ^]^ This liver‐specific gene signaling correlated with adverse prognosis. More importantly, knockdown of TRIB3 successfully accentuated sorafenib's therapeutic effect in vivo. This highlights the potential of TRIB3 as a prognostic biomarker and a therapeutic target in sorafenib treatment. This was further supported by our findings that such elevated TRIB3 expression in tumors was positively correlated with increased intrahepatic metastasis and vascular invasion, as well as early recurrence in patients receiving sorafenib therapy.

Notably, TRIB3 expression was relatively low in primary HCC tumors but aberrantly increased upon sorafenib therapy in HCC patients. In fact, whether in mouse hepatoma models (either orthotopic or spontaneous), or in vivo culture of hepatoma cells, sorafenib administration markedly elevated TRIB3 expression. We demonstrated that TRIB3 was triggered by sorafenib‐elicited ROS‐ER stress axis in HCC, which was consistent with the identity of TRIB3 as a stress sensor. Correspondingly, interruption of ROS‐ER stress axis significantly abrogated sorafenib‐induced TRIB3 upregulation and subsequent hepatoma growth and lung metastasis in vivo. These findings reveal an intrinsic paradoxical role of sorafenib, which induces protumorigenic TRIB3 during its effective targeting of tumors, eventually limiting it clinical efficacy.

Despite the oncogenic effects of TRIB3 in multiple malignancies, how TRIB3 is involved in therapeutic resistance remains poorly understood.^[^ ^23^, ^24^, ^25^, ^26^ ^]^ Interestingly, our results indicated that neither overexpression nor knockout of TRIB3 affected EMT process or migration in hepatoma cells in vitro, although TRIB3 deficiency successfully suppressed sorafenib‐induced lung metastasis in vivo, emphasizing the essential role of in vivo microenvironment in TRIB3‐mediated sorafenib resistance. More precisely, our results provide evidence that elevated TRIB3 promoted tumor growth, angiogenesis, and metastasis via facilitating the selective enrichment of neutrophils, as indicated by the following observations. First, a selective increase in Gr1^+^ neutrophils, but not other subsets of lymphocytes or myeloid cells, was detected in TRIB3‐overexpressing mouse hepatoma. Second, neutrophils abrogated sorafenib‐elicited inhibition of proliferation, EMT, and pro‐angiogenic activity of hepatoma cells. Third, neutrophil depletion effectively attenuated TRIB3‐driven aggressive angiogenesis and the enhanced EMT process in hepatoma, and thereby augment the anti‐tumor efficacy of sorafenib treatment. Therefore, sorafenib‐induced TRIB3 triggered secondary resistance via remodeling the tumor immune microenvironment. Analogously, recent studies have shown that TRIB3 dampens CD8^+^ T cell infiltration by repressing the STAT1‐CXCL10 axis and consequently induces immune evasion in colorectal cancer.^[^ ^22^ ^]^ Thus, targeting TRIB3 can serve as a potential approach to remodel the constitution of tumor immune microenvironment and to sensitize tumors to various therapeutic strategies.

Neutrophils, a key component of the tumor microenvironment,^[^ ^42^, ^43^ ^]^ played a pivotal role in TRIB3‐elicited sorafenib resistance. Mechanistically, TRIB3‐amplified NF‐κB signaling upregulates CXCR1/2 ligands in hepatoma cells, attracting neutrophils that enhance EMT via the OSM‐STAT3 pathway, thereby diminishing sorafenib's efficacy. Inhibiting NF‐κB signaling or neutralizing OSM significantly augmented the therapeutic efficacy of sorafenib and successfully led to sustained cancer regression in vivo. These findings shed light on the intricate interplay between sorafenib treatment and the tumor microenvironment, revealing a previously unrecognized feedback loop that drives therapeutic resistance.

The current study provided a comprehensive evaluation of sorafenib‐elicited TRIB3 in secondary resistance to sorafenib in HCC. TRIB3, which is triggered by sorafenib‐elicited ROS‐ER stress axis, facilitates the neutrophil recruitment by upregulating the expression of CXCR1/2 ligands through boosting NF‐κB signaling. Thereafter, these accumulated neutrophils in turn enhance EMT process in hepatoma cells via OSM‐STAT3 pathway and eventually convert the antitumorigenic efficacy of sorafenib to lung metastasis. Our investigation establishes an innovative connection between sorafenib‐induced intrinsic oncogenic alterations in malignant cells and remodeling of immune microenvironment in the context of secondary resistance to sorafenib. In addition to its biological importance, our work may be relevant in clinical management of cancer patients. Our findings not only support the clinical evaluation of TRIB3 as a biomarker to predict a patient's response to sorafenib treatment, but also provide a new clue for optimizing HCC therapy. We suggest that combinational treatment by sorafenib and blockade of TRIB3‐related signaling may be a more effective therapy for patients with advanced HCC. Although specific TRIB3 inhibitors are not yet available, recent studies have identified several compounds with TRIB3 inhibitory properties, including hesperidin.^[^ ^44^ ^]^ In line with our findings, these compounds demonstrated ability to inhibit tumor progression by inducing ferroptosis in preclinical models. Furthermore, our preclinical investigations also confirmed that targeting PERK or CXCR1/2 pathways, which are involved in the induction and functional mechanisms of TRIB3, can enhance the therapeutic efficacy of sorafenib. Therefore, TRIB3‐related pathways may offer significant promise for the future clinical treatment of HCC and other malignancies.

## 4 Experimental Section

### Patients and Specimens

196 patients pathologically diagnosed with primary hepatocellular carcinoma at the Cancer Center of Sun Yat‐sen University were included in this study (Tables S1 and S2, Supporting Information). None of the patients had received any anticancer therapy prior to the initial sampling, and those with concurrent autoimmune disease, human immunodeficiency virus, or syphilis were excluded. An additional cohort of 30 HCC patients, who had undergone primary surgical intervention and subsequently experienced disease relapse, was selected for a second round of sampling. (Table S3, Supporting Information). Clinical stages were classified according to the guidelines of the Union for International Cancer Control. All samples were anonymously coded in accordance with local ethical guidelines (as stipulated by the Declaration of Helsinki). Written informed consent was obtained from the patients, and the protocol was approved by the Ethical Review Board of Sun Yat‐sen University (ethical official number: GZR2018‐166).

### Animal Experiments

Wild‐type female C57BL/6 mice (6 weeks old) were obtained from the Guangdong Medical Laboratory Animal Center (GDMLAC‐07, Guangzhou, China). These mice were housed under specific pathogen‐free conditions in the animal facilities of the Cancer Center of Sun Yat‐sen University and were randomly assigned to experimental groups. Animal experiments were performed with the approval of the Institutional Animal Care and Use Committee of Sun Yat‐sen University (SYSU‐IACUC‐2022‐000686). The individual mouse was considered the experimental unit within the studies. All mice were randomly grouped and no animal data were excluded. Hepa1‐6 hepatoma was established as detailed in Figures 1, 2, and 6H and Figures S1C, S3B, S4D,F, and S5D,H (Supporting Information). Spontaneous hepatoma was induced via hydrodynamic injection as described previously.^[^ ^45^ ^]^ Briefly, plasmids encoding myr‐AKT1 (20 µg) and N‐RasG12V (20 µg), along with sleeping beauty transposase (1.6 µg), were diluted in 2 ml saline and injected into the lateral tail vein of C57BL/6 mice in 5 to 7 secs. Tumor volumes were calculated using the formula: Volume = Length × (Width)^2^/2. For the detection of lung metastasis, haematoxylin and eosin (H&E) staining of lung tissues was performed. Briefly, lung tissues from hepatoma‐bearing mice were excised, fixed in formalin, and then embedded in paraffin. The embedded lungs were systematically sectioned at a thickness of 3.5 µm, with an 80 µm spacing between each section. A total of 30 sections were stained with H&E. The number of metastatic foci in each section was assessed by two researchers who were blinded to the treatment. The average number of metastatic foci from these 30 sections was recorded. For bioluminescence imaging, 150 mg kg^−1^ of D‐luciferin (GOLDBIO) was intravenously administered to luciferase‐labeled hepatoma‐bearing mice. Following injection, the mice were anesthetized with isoflurane and imaged using the IVIS Spectrum in vivo imaging system (PerkinElmer), in accordance with the manufacturer's instructions. Imaging data were quantified using Living Image software (Caliper Life Sciences).

### Cell Lines and Preparation of Tumor Culture Supernatant (TSN)

Human Hep G2 cells, SNU‐449, and mouse Hepa1‐6 cells were obtained from the American Type Culture Collection (HB‐8065; CRL‐2234; CRL‐12461), and human Huh‐7 cells and MHCC‐97H cells were obtained from Shanghai Institutes for Biological Sciences (SCSP‐526; SCSP‐5092). Cells were tested for mycoplasma contamination using a single‐step PCR method. All of these cell lines were maintained in a culture medium (CM) composed of DMEM supplemented with 10% FBS (Gibco). TSN was prepared as described previously.^[^ ^46^ ^]^ In brief, 5 × 10^6^ tumor cells were plated in 10 ml of complete DMEM medium in 100‐mm dishes for 24 h. Subsequently, the medium was replaced with complete medium containing 10% FBS. After 2 days, the supernatant was centrifuged and stored. In some experiments, hepatoma cells were pre‐treated with 10 µm sorafenib for 48 h before harvesting the supernatants.

### Immunohistochemistry and Immunofluorescence

Paraffin‐embedded human HCC or mouse hepatoma samples were cut in 5‐µm sections and processed for immunohistochemistry. The sections were subsequently incubated with antibodies against human TRIB3, CD15, CD34, or antibodies against mouse B220, CD4, CD8, F4/80, Gr1, Nkp46, Ki67, and CD31, followed by staining using the Envision System (Dako). Immunohistochemical evaluation was conducted by two independent observers who were blinded to the clinical outcome. At a low‐power field (×100), the tissue sections were screened, and the 5 most representative fields were selected using a Leica DM4 B inverted research microscope (Leica, Germany). Thereafter, respective areas were measured at ×400 magnification (0.146 mm^2^ per field). For CD15, B220, CD4, CD8, F4/80, Gr1, Nkp46, and Ki67, only cells with a clearly visible nucleus (marked by hematoxylin staining) and a brown DAB staining signal were defined as positive cells. Furthermore, positively signals that were smaller than the size of circulating immune cells (5 µm) were excluded from counting. For microvessel density, single endothelial cells or clusters of endothelial cells positive for CD31 were considered as individual vessels. The average of counts by two investigators was applied in the following analysis to minimize interobserver variability. Microvessel area and integrated TRIB3 density was determined by IMMAGE J software.

For immunofluorescence analysis of cultured cells, cells grown on a cover slide were fixed, permeabilized, and incubated with rabbit anti‐human TRIB3 followed by incubation with Alex Fluor 555‐conjugated anti‐rabbit IgG. Nuclei were counterstained with DAPI. Immunofluorescence staining images were visualized by confocal microscopy (LSM880 with Fast airy scan, Carl Zeiss). The antibodies used in immunohistochemistry/immunofluorescence are shown in Table S4 (Supporting Information).

### Immunoblotting

Cells from in vitro culture system or tissue samples were washed three times with PBS, and the resulting pellets were resuspended in lysis buffer for 20 min on ice. After centrifugation at 10 000g for 10 min, the supernatants were dissolved in Laemmli sample buffer and heated at 95 °C for 5 min. Equal amounts of total protein were then separated by 10% SDS‐polyacrylamide gel electrophoresis and transferred onto nitrocellulose membranes. The membranes were blocked with 3% bovine serum albumin, and the presence of indicated proteins on the blots was detected using specific antibodies and a commercial ECL kit. The antibodies used in immunoblotting are shown in Table S5 (Supporting Information).

### Leukocyte Isolation from Tissues and Preparation of CM from Tumor Leukocytes

Fresh HCC biopsy specimens were cut into small pieces and digested in RPMI 1640 supplemented with 0.05% collagenase IV (Sigma–Aldrich), 0.002% DNase I (Roche), and 20% FBS at 37 °C for 30 min. Dissociated cells were filtered through a 150‐µm mesh, thereafter tissue mononuclear leukocytes and neutrophils were isolated using ficoll density gradient centrifugation. The mononuclear leukocytes were further harvested and the tumor‐infiltrating macrophages, T cells, NK cells, and B cells were sorted by FACS according to CD14, CD3, CD56, and CD19 expression, while neutrophil were purified by magnetic‐activated cell sorting. For preparation of CM from tumor leukocytes, 1 × 10^6^ sorted cells were resuspended in 1 ml of CM and cultured. After 24 h, the supernatants were harvested.

### Neutrophil Isolation and Migration Assay

Human neutrophils were isolated from peripheral blood of healthy donors using density‐gradient separation on Polymorphprep. The pale‐red granulocyte layer was collected, washed, and subjected to brief hypotonic lysis to remove contaminating erythrocytes. Neutrophils were resuspended in DMEM medium. 100 µL neutrophil suspension (2.5 × 10^6^/mL) in DMEM was added to the upper chamber of each 0.2 µm fibronectin‐coated polycarbonate well inserts of a 24‐well Transwell system (6.5‐mm diameter, 5‐µm pores), and the plate was incubated at 37 °C for 5 h. Neutrophils that migrated to the lower chamber and those detached from the lower surface of the filters through gentle washing were harvested and counted. Migration assays were conducted with DMEM medium supplemented with 10% FBS or culture supernatants from hepatoma cells in the lower chamber of the Transwell system.

### Cell Viability Assay

Approximately 8000 cells per well in 500 µL of medium were seeded in a 24‐well plate, cultured overnight, and subsequently treated with DMSO or sorafenib, with or without the presence of tumor neutrophils in the upper chamber of a Transwell plate for varying durations. Following treatment, CCK‐8 solution (Table S6, Supporting Information) was then added to each well, and the cells were further incubated at 37 °C for 1 h. The absorbance of 450 nm was measured and normalized to the untreated cells.

### Tumor Cell Migration Assay

The migration assay was performed in a 24‐well Boyden chamber with an 8 µm polycarbonate membrane. Huh‐7 or Hepa1‐6 cells were treated with DMSO or sorafenib in the absence or presence of conditioned medium from neutrophil cultures (N‐CM). Sometimes, isotype or OSM neutralizing antibody were add to the N‐CM before treating Huh‐7 cells. Thereafter, cells (3 × 10^4^) in 100 µL of serum‐free DMEM were added to the upper compartment of the chamber, while the lower chamber was filled with 500 µl of DMEM containing 10% FBS. After 10 h of incubation, the cells remaining on the upper surface of the membrane were removed. The migrated tumor cells on the lower surface of the membrane were washed with PBS, fixed, stained with crystal violet, and counted under a light microscope (Leica, DM4 B).

### ROS Analysis

Huh‐7 cell in PBS were incubated with 2.5 µm DCFH‐DA at 37 °C for 15 min in a humidified atmosphere of 5% CO_2_ in air. Following incubation, untreated and 10 µm sorafenib‐treated Huh‐7 cell were immediately evaluated for ROS levels using BD FACS Calibur flow cytometer and FlowJo_v10 software.

### Polymerase Chain Reaction

Trizol reagent (Invitrogen) was used to isolate total RNA of cells from tissue or in vitro culture system. Aliquots (2 µg) of the RNA were reverse transcribed using Moloney murine leukemia virus reverse transcriptase (Promega). The specific primers used to amplify the genes are listed in Table S7 (Supporting Information). The real time PCR was performed in triplicate using Hieff qPCR SYBR Green Master Mix in a Roche LightCycler 480 System. For high fidelity polymerase chain reaction, a KOD One PCR Master Mix (Toyobo, KMM‐201) was used.

### Regulation of TRIB3 Expression in Cancer Cells

Hepatoma cells were untreated or stimulated with sorafenib for 48 h. In some experiments, before exposure to sorafenib, cells were pretreated with PERK inhibitor (GSK2606414) or ROS scavenger (NAC) for 30 min. Thereafter, cells were subject to RT‐PCR and WB to detect TRIB3.

### Construction of Gene‐Deficient or Gene‐Overexpressing Cells

To establish the knockdown of TRIB3 in Huh‐7 cells or Rela in Hepa1‐6 cells, specific shRNA targeting TRIB3 or Rela were cloned into the pLKO‐puro vector (Sigma–Aldrich). To generate stable cell lines overexpressing C‐terminal‐tagged TRIB3 in Huh‐7 cells and Hepa1‐6 cells, human and mouse TRIB3 genes were amplified by PCR from cDNA, verified by DNA sequencing, and inserted into the pCDH‐CMV‐MCS‐3xFLAG‐EF1‐copGFP‐T2A‐Puro (Ruibiotech). Thereafter, the plasmids were transfected into HEK293T cells together with psPAX2 (Addgene, 12 260) and pVSV‐G (Addgene, 138 479) using polyethyleneimine (Sigma–Aldrich, 408 727) to generate the lentiviral particles. After 2 days, the lentiviral particles were harvested and used to infect hepatoma cells in the presence of 8 µg mL^−1^ polybrene (Sigma–Aldrich). For construction of Rela^SH^Trib3^OE^ Hepa1‐6 cells, Rela^SH^ Hepa1‐6 cells were re‐infected with Trib3^OE^ lentiviral particles, and GFP^+^ cells were sorted using fluorescence‐activated cell sorting to abtain Rela^SH^Trib3^OE^ Hepa1‐6 cells.

For the deletion of Trib3 in Hepa1‐6 cells, paired sgRNAs targeting Trib3 were designed and synthesized by IGE Biotechnology. Essentially, the paired sgRNAs were subsequently inserted into pSpCas9 (BB)‐2A‐GFP (PX458, IGE Biotechnology). PX458 was transfected into Hepa1‐6 cells using Lipofectamine 3000. After 48 h, single GFP‐positive Hepa1‐6 cells were sorted using FACS and cultured individually for further screening to confirm the successful genome editing.

### ELISA

100 µL of conditioned cell culture media were added to a high binding 96 well microplate that was incubated overnight with indicated capture antibody. ELISA were performed following the manufacturer's instructions.

### Dual Luciferase Assays

293T cells were co‐transfected with luciferase constructs (pGL3) containing TRIB3 promoter sequences (−1265 to +609), constructs (pCDH‐CMV‐3X Flag) encoding CHOP or ATF4 and encoding Renilla construct (pRL‐TK) as the control. After 48 h of transfection, cells were lysed with Passive Lysis Buffer. The activities of Firefly and Renilla luciferase in the lysates were measured with the Dual Luciferase Reporter Gene Assay Kit (Yeasen). The final results were normalized to Renilla luciferase activity.

### Statistical Analysis

Results are expressed as means ± SEM. All data were analyzed using two tailed tests unless otherwise specified. For data normally distributed, the Student's t test was applied. For multiple comparisons, an analysis of variance followed by with Tukey's correction was applied. All statistical tests were performed with GraphPad Prism (v.6) software. Cumulative survival time was calculated by the Kaplan‐Meier method, and survival was measured in months from resection to dead or the last review. The log‐rank test was applied to compare the groups. *P* < 0.05 was considered statistically significant. All data acquired from animal models were used in analyses.

## Conflict of Interest

The authors declare no conflict of interest.

## Author Contributions

X.‐Y.W., Y.L., and R.‐Q.W. contributed equally to this work. X.‐Y.W. and R.‐Q.W. performed most of the experiments and analyzed the results. X.‐M.L. and Y.L. provided clinical samples and analyzed the related clinical data. Y.‐T.L., Y.‐Z.W., and Y.‐Q.X. contributed to analyze the data and guided computational analysis. D.‐M.K., J.X., Z.‐L.Z, and K.H. contributed to study design, supervised the study, and contributed to writing the manuscript. The order of the co‐first authors was assigned based on their efforts and contributions to the study.

## Supporting information



Supporting Information

## Data Availability

The microarray experiment investigating the effect of sorafenib on hepatocellular carcinoma cell lines was performed by other study, with the data accessible in the GEO database under number accession GSE186280. Additionally, the previously published scRNA‐seq datasets of primary HCC patients, which were reanalyzed in this study, can be accessed from the GEO database under accession number GSE125449. All analysis and figures were generated using publicly available software packages. No custom code was used in this study. All other raw data are available from the corresponding authors upon request.

## References

[1] Z. Sas , E. Cendrowicz , I. Weinhäuser , T. P. Rygiel , Int. J. Mol. Sci. 2022, 23, 3778. 35409139 10.3390/ijms23073778 PMC8998420

[2] A. J. Craig , J. von Felden , T. Garcia‐Lezana , S. Sarcognato , A. Villanueva , Nat. Rev. Gastroenterol. Hepatol. 2020, 17, 139. 31792430 10.1038/s41575-019-0229-4

[3] Y. B. Yang , C. Y. Wu , X. Y. Wang , J. Deng , W. J. Cao , Y. Z. Tang , C. C. Wan , Z. T. Chen , W. Y. Zhan , H. Shan , D. M. Kuang , Y. Wei , Mol. Ther. 2023, 31, 105. 36183166 10.1016/j.ymthe.2022.09.019 PMC9840147

[4] C. F. A. Ramirez , D. Taranto , M. Ando‐Kuri , M. H. P. de Groot , E. Tsouri , Z. Huang , D. de Groot , R. J. C. Kluin , D. J. Kloosterman , J. Verheij , J. Xu , S. Vegna , L. Akkari , Nat. Commun. 2024, 15, 2581. 38519484 10.1038/s41467-024-46835-2 PMC10959959

[5] D. M. Kuang , X. Xiao , Q. Zhao , M. M. Chen , X. F. Li , R. X. Liu , Y. Wei , F. Z. Ouyang , D. P. Chen , Y. Wu , X. M. Lao , H. Deng , L. Zheng , J. Clin. Invest. 2014, 124, 4657. 25244097 10.1172/JCI74381 PMC4191045

[6] C. Yang , H. Zhang , L. Zhang , A. X. Zhu , R. Bernards , W. Qin , C. Wang , Nat. Rev. Gastroenterol. Hepatol. 2023, 20, 203. 36369487 10.1038/s41575-022-00704-9

[7] W. Leowattana , T. Leowattana , P. Leowattana , World J. Gastroenterol. 2023, 29, 1551. 36970588 10.3748/wjg.v29.i10.1551 PMC10037251

[8] S. M. Wilhelm , C. Carter , L. TanL , D. Wilkie , A. McNabola , H. Rong , C. Chen , X. Zhang , P. Vincent , M. McHugh , Y. Cao , J. Shujath , S. Gawlak , D. Eveleigh , B. Rowley , L. Liu , L. Adnane , M. Lynch , D. Auclair , I. Taylor , R. Gedrich , A. Voznesensky , B. Riedl , L. E. Post , G. Bollag , P. A. Trail , Cancer Res. 2004, 64, 7099. 15466206 10.1158/0008-5472.CAN-04-1443

[9] S. Tanaka , S. Arii , Semin. Oncol. 2012, 39, 486. 22846865 10.1053/j.seminoncol.2012.05.005

[10] J. M. Llovet , S. Ricci , V. Mazzaferro , P. Hilgard , E. Gane , J. F. Blanc , A. C. de Oliveira , A. Santoro , J. L. Raoul , A. Forner , M. Schwartz , C. Porta , S. Zeuzem , L. Bolondi , T. F. Greten , P. R. Galle , J. F. Seitz , I. Borbath , D. Häussinger , T. Giannaris , M. Shan , M. Moscovici , D. Voliotis , J. Bruix , N. Engl. J. Med. 2008, 359, 378. 18650514 10.1056/NEJMoa0708857

[11] A. L. Cheng , Y. K. Kang , Z. Chen , C. J. Tsao , S. Qin , J. S. Kim , R. Luo , J. Feng , S. Ye , T. S. Yang , J. Xu , Y. Sun , H. Liang , J. Liu , J. Wang , W. Y. Tak , H. Pan , K. Burock , J. Zou , D. Voliotis , Z. Guan , Lancet Oncol. 2009, 10, 25. 19095497 10.1016/S1470-2045(08)70285-7

[12] J. Bruix , J. L. Raoul , M. Sherman , V. Mazzaferr , L. Bolondi , A. Craxi , P. R. Galle , A. Santoro , M. Beaugrand , A. Sangiovanni , C. Porta , G. Gerken , J. A. Marrero , A. Nadel , M. Shan , M. Moscovici , D. Voliotis , J. M. Llovet , J. Hepatol. 2012, 57, 821. 22727733 10.1016/j.jhep.2012.06.014 PMC12261288

[13] H. van Malenstein , J. Dekervel , C. Verslype , E. Van Cutsem , P. Windmolders , F. Nevens , J. van Pelt , Cancer Letters. 2013, 329, 74. 23111106 10.1016/j.canlet.2012.10.021

[14] M. Mu , C. X. Huang , C. Qu , P. L. Li , X. N. Wu , W. Yao , C. Shen , R. Huang , C. C. Wan , Z. W. Jian , L. Zheng , R. Q. Wu , X. M. Lao , D. M. Kuang , Cancer Res. 2024, 84, 841. 38231484 10.1158/0008-5472.CAN-23-1796

[15] R. Dadu , S. G. Waguespack , S. I. Sherman , M. I. Hu , N. L. Busaidy , C. Jimenez , M. A. Habra , A. K. Ying , R. L. Bassett , M. E. Cabanillas , The Oncologist. 2014, 19, 477. 24733667 10.1634/theoncologist.2013-0409 PMC4012968

[16] M. Hendrixson , Y. Gladkiy , A. Thyagarajan , R. P. Sahu , Med. Sci. (Basel) 2024, 12, 20. 38651414 10.3390/medsci12020020 PMC11036230

[17] M. Oya , S. Kaneko , T. Imai , T. Tsujino , T. Sunaya , Y. Okayama , Cancer Chemother. Pharmacol. 2022, 89, 761. 35445315 10.1007/s00280-022-04428-0 PMC9135823

[18] E. Kiss‐Toth , S. M. Bagstaff , H. Y. Sung , V. Jozsa , C. Dempsey , J. C. Caunt , K. M. Oxley , D. H. Wyllie , T. Polgar , M. Harte , L. A. O'neill , E. E. Qwarnstrom , S. K. Dower , J. Biol. Chem. 2004, 279, 42703. 15299019 10.1074/jbc.M407732200

[19] P. A. Eyers , K. Keeshan , N. Kannan , Trends Cell Biol. 2017, 27, 284. 27908682 10.1016/j.tcb.2016.11.002 PMC5382568

[20] M. Ruiz‐Cantos , C. E. Hutchison , C. C. Shoulders , Cancers (Basel) 2021, 13, 4517. 34572744 10.3390/cancers13184517 PMC8467127

[21] A. J. Bowers , S. Scully , J. F. Boylan , Oncogene 2003, 22, 2823. 12743605 10.1038/sj.onc.1206367

[22] S. Shang , Y. W. Yang , F. Chen , L. Yu , S. H. Shen , K. Li , B. Cui , X. X. Lv , C. Zhang , C. Yang , J. Liu , J. J. Yu , X. W. Zhang , P. P. Li , S. T. Zhu , H. Z. Zhang , F. Hua , Sci. Transl. Med. 2022, 14, eabf0992. 34985967 10.1126/scitranslmed.abf0992

[23] J. J. Yu , D. D. Zhou , X. X. Yang , B. Cui , F. W. Tan , J. Wang , K. Li , S. Shang , C. Zhang , X. X. Lv , X. W. Zhang , S. S. Liu , J. M. Yu , F. Wang , B. Huang , F. Hua , Z. W. Hu , Nat. Commun. 2020, 11, 3660. 32694521 10.1038/s41467-020-17385-0 PMC7374170

[24] A. Jaiswal , K. Murakami , A. Elia , Y. Shibahara , S. J. Done , S. A. Wood , N. J. Donato , P. S. Ohashi , M. Reedijk , Proc. Natl. Acad. Sci. USA 2021, 118, e2101592118. 34518219 10.1073/pnas.2101592118 PMC8463885

[25] K. Li , F. Wang , Z. N. Yang , B. Cui , P. P. Li , Z. Y. Li , Z. W. Hu , H. H. Zhu , Theranostics 2020, 10, 10326. 32929351 10.7150/thno.45924 PMC7481410

[26] K. Li , F. Wang , Z. N. Yang , T. T. Zhang , Y. F. Yuan , C. X. Zhao , Z. Yeerjiang , B. Cui , F. Hua , X. X. Lv , X. W. Zhang , J. J. Yu , S. S. Liu , J. M. Yu , S. Shang , Y. Xiao , Z. W. Hu , Nat. Commun. 2020, 11, 6316. 33298911 10.1038/s41467-020-20107-1 PMC7725785

[27] X. Chen , C. Shi , M. He , S. Xiong , X. Xia , Signal Transduct. Target. Ther. 2023, 8, 352. 37709773 10.1038/s41392-023-01570-w PMC10502142

[28] N. Ohoka , S. Yoshii , T. Hattori , K. Onozaki , H. Hayashi , EMBO J. 2005, 24, 1243. 15775988 10.1038/sj.emboj.7600596 PMC556400

[29] X. Sun , Z. Ou , R. Chen , X. Niu , D. Chen , R. Kang , D. Tang , Hepatology 2016, 63, 173. 26403645 10.1002/hep.28251 PMC4688087

[30] Z. J. Li , H. Q. Dai , X. W. Huang , J. Feng , J. H. Deng , Z. X. Wang , X. M. Yang , Y. J. Liu , Y. Wu , P. H. Chen , H. Shi , J. G. Wang , J. Zhou , G. D. Lu , Acta Pharmacol. Sin. 2021, 42, 301. 32699265 10.1038/s41401-020-0478-3 PMC8026986

[31] A. Capucetti , F. Albano , R. Bonecchi , Front. Immunol. 2020, 11, 1259. 32733442 10.3389/fimmu.2020.01259 PMC7363767

[32] M. Metzemaekers , M. Gouwy , P. Proost , Cell. Mol. Immunol. 2020, 17, 433. 32238918 10.1038/s41423-020-0412-0 PMC7192912

[33] K. Kohli , V. G. Pillarisetty , T. S. Kim , Cancer Gene Ther. 2022, 29, 10. 33603130 10.1038/s41417-021-00303-x PMC8761573

[34] G. Niu , K. L. Wright , M. Huang , L. Song , E. Haura , J. Turkson , S. Zhang , T. Wang , D. Sinibaldi , D. Coppola , R. Heller , L. M. Ellis , J. Karras , J. Bromberg , D. Pardoll , R. Jove , H. Yu , Oncogene 2002, 21, 2000. 11960372 10.1038/sj.onc.1205260

[35] J. Yuan , F. Zhang , R. Niu , Sci. Rep. 2015, 5, 17663. 26631279 10.1038/srep17663 PMC4668392

[36] W. Jin , Cells 2020, 9, 217.31952344

[37] M. Ringelhan , D. Pfister , T. O'Connor , E. Pikarsky , M. Heikenwalder , Nat. Immunol. 2018, 19, 222. 29379119 10.1038/s41590-018-0044-z

[38] L. X. Yu , Y. Ling , H. Y. Wang , NPJ Precis. Oncol. 2018, 2, 6. 29872724 10.1038/s41698-018-0048-z PMC5871907

[39] M. Kotsari , V. Dimopoulou , J. Koskinas , A. Armakolas , Int. J. Mol. Sci. 2023, 24, 11471. 37511228 10.3390/ijms241411471 PMC10380581

[40] D. M. Kuang , Q. Zhao , J. Xu , J. P. Yun , C. Wu , L. Zheng , J. Immunol. 2008, 181, 3089. 18713979 10.4049/jimmunol.181.5.3089

[41] R. Q. Wu , X. M. Lao , D. P. Chen , H. Qin , M. Mu , W. J. Cao , J. Deng , C. C. Wan , W. Y. Zhan , J. C. Wang , L. Xu , M. S. Chen , Q. Gao , L. Zheng , Y. Wei , D. M. Kuang , Immunity 2023, 56, 180. 36563676 10.1016/j.immuni.2022.11.014

[42] R. Xue , Q. Zhang , Q. Cao , R. Kong , X. Xiang , H. Liu , M. Feng , F. Wang , J. Cheng , Z. Li , Q. Zhan , M. Deng , J. Zhu , Z. Zhang , N. Zhang , Nature 2022, 612, 141. 36352227 10.1038/s41586-022-05400-x

[43] D. M. Kuang , Q. Zhao , Y. Wu , C. Peng , J. Wang , Z. Xu , X. Y. Yin , L. Zheng , J. Hepatol. 2011, 54, 948. 21145847 10.1016/j.jhep.2010.08.041

[44] L. Chen , W. Lin , H. Zhang , S. Geng , Z. Le , F. Wan , Q. Huang , H. Chen , X. Liu , J. J Lu , L. Kong , Cell Death Dis. 2024, 15, 178. 38429254 10.1038/s41419-024-06472-5 PMC10907716

[45] C. X. Huang , X. M. Lao , X. Y. Wang , Y. Z. Ren , Y. T. Lu , W. Shi , Y. Z. Wang , C. Y. Wu , L. Xu , M. S. Chen , Q. Gao , L. Liu , Y. Wei , D. M. Kuang , Cancer Cell 2024, 42, 2082. 39547231 10.1016/j.ccell.2024.10.012

[46] D. M. Kuang , C. Peng , Q. Zhao , Y. Wu , M. S. Chen , L. Zheng , Hepatology 2010, 51, 154. 19902483 10.1002/hep.23291

