# Supplementary material for: Tribbles Pseudokinase 3 Converts Sorafenib Therapy to Neutrophil‐Mediated Lung Metastasis in Hepatocellular Carcinoma
Source: Adv Sci (Weinh). 2025 Feb 11;12(13):2413682. doi: 10.1002/advs.202413682 (PMC11967757; doi:10.1002/advs.202413682)
Supplement: Supplementary file 1 — Supporting Information [file ADVS-12-2413682-s001.pdf]

ADVANCED SCIENCE
Open Access

Supporting Information

for *Adv. Sci.*, DOI 10.1002/advs.202413682

Tribbles Pseudokinase 3 Converts Sorafenib Therapy to Neutrophil-Mediated Lung Metastasis in Hepatocellular Carcinoma

Xu-Yan Wang, Yuan Liao, Rui-Qi Wang, Yi-Tong Lu, Ying-Zhe Wang, Yu-Qi Xin, Dong-Ming Kuang, Xiang-Ming Lao*, Junying Xu*, Zhi-Ling Zhou* and Kunhua Hu**

Supporting Information

Tribbles Pseudokinase 3 Converts Sorafenib Therapy to Neutrophil-Mediated Lung Metastasis in Hepatocellular Carcinoma

Xu-Yan Wang, *Yuan Liao*, *Rui-Qi Wang*, *Yi-Tong Lu*, *Ying-Zhe Wang*, *Yu-Qi Xin*, *Dong-Ming Kuang*,* *Xiang-Ming Lao*,* *Junying Xu*,* *Zhi-Ling Zhou*,* and *Kunhua Hu**

Table of Contents

Supplementary Figures

Figure S1 Inducing TRIB3 by sorafenib promotes therapeutic resistance and metastasis.
Figure S2 TRIB3 is triggered by sorafenib-elicited ROS-ER stress axis in HCC.
Figure S3 In vivo milieus dictate TRIB3's protumorigenic properties.
Figure S4 Neutrophils are essential for TRIB3-driven pro-tumorigenesis.
Figure S5 Elevated TRIB3 attracts neutrophils through boosting NF-κB-CXCL signaling.
Figure S6 Neutrophils suppress therapeutic efficacy of sorafenib via OSM/STAT3 axis.

Supplementary Tables

Table S1 Clinical characteristics of 196 hepatocellular carcinoma patients.
Table S2 Characteristics of 14 patients contributing fresh samples.
Table S3 Characteristics of 30 patients contributing twice samples in Figure 1B and C.
Table S4 Antibodies for immunohistochemistry, immunofluorescence and flow cytometry.
Table S5 Antibodies for immunoblotting.
Table S6 Recombinant proteins, peptides, chemicals, and critical commercial assays.
Table S7 Sequence-based reagents.

Supplementary Figures

Figure S1

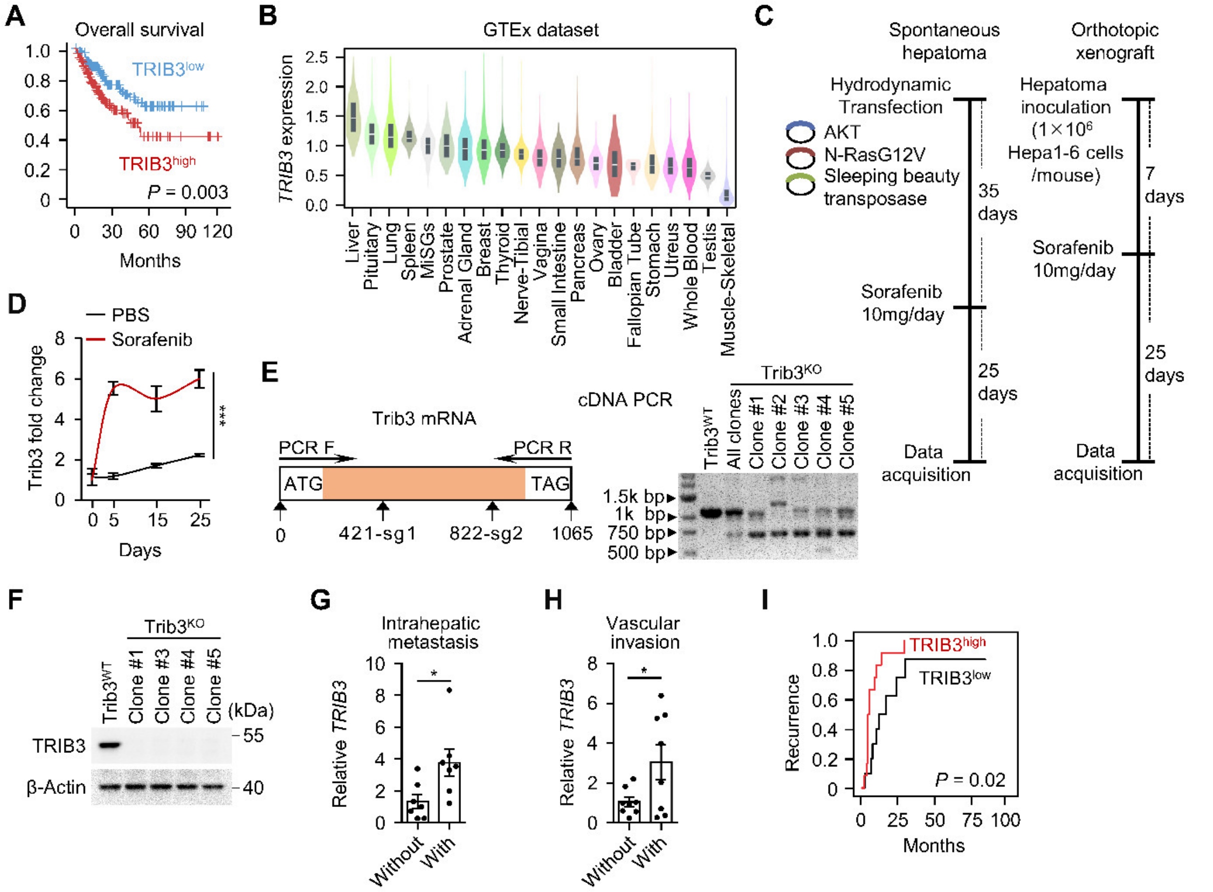

Figure S1. Inducing TRIB3 by sorafenib promotes therapeutic resistance and metastasis. A) A total of 291 HCC samples with complete follow-up data from TCGA dataset were divided into two groups according to the median value of *TRIB3* expression: TRIB3^{low} (n = 146, blue) and TRIB3^{high} (n = 145, red). Cumulative overall survival time were calculated using the Kaplan-Meier method and analyzed by the log-rank test. **B**) The expression of *TRIB3* in 21 human major tissues from Genotype-Tissue Expression (GTEx) project. **C**) Orthotopic xenograft and spontaneous hepatoma in C57BL/6 mice were induced by orthotopic inoculation of Hepa1-6 cells and hydrodynamic tail vein transfection of activation forms of AKT and N-RasG12V proto-oncogenes, respectively. Thereafter, the mice were treated with sorafenib or equivalent PBS as described. **D)** Hepa1-6 hepatoma-bearing mice were injected with PBS or sorafenib. The expression of Trib3 in the tumor tissues were dynamically assessed by RT-PCR (n = 5). **E,F**) Trib3-knockout in Hepa1-6 cells was performed by transducing with PX458 plasmid containing dual small-guide RNA (sgRNA). The efficacy of TRIB3 knockout was determined by cDNA PCR (**E**) and immunoblotting (**F**), respectively. Clones 1, 3-5 were mixed for establishing Hepa1-6 HCC mouse model. **G,H**) Correlations of *TRIB3* expression with intrahepatic metastasis (**G**, n = 14) and vascular invasion (**H**, n = 16) in patients with HCC. **I**) Associations of *TRIB3* expression in tumor with patients' response to sorafenib therapy. 22 HCC patients from TCGA dataset, who received sorafenib treatment after surgery, were divided into two groups: TRIB3^{low} (n = 10, black) and TRIB3^{high} (n = 12, red). Recurrence time were calculated using the Kaplan-Meier method and analyzed by the log-rank test. Data represent mean ± SEM of three independent experiments. **p* <0.05, ***p< 0.001, Student's *t* test (**G** and **H**).

Figure S2

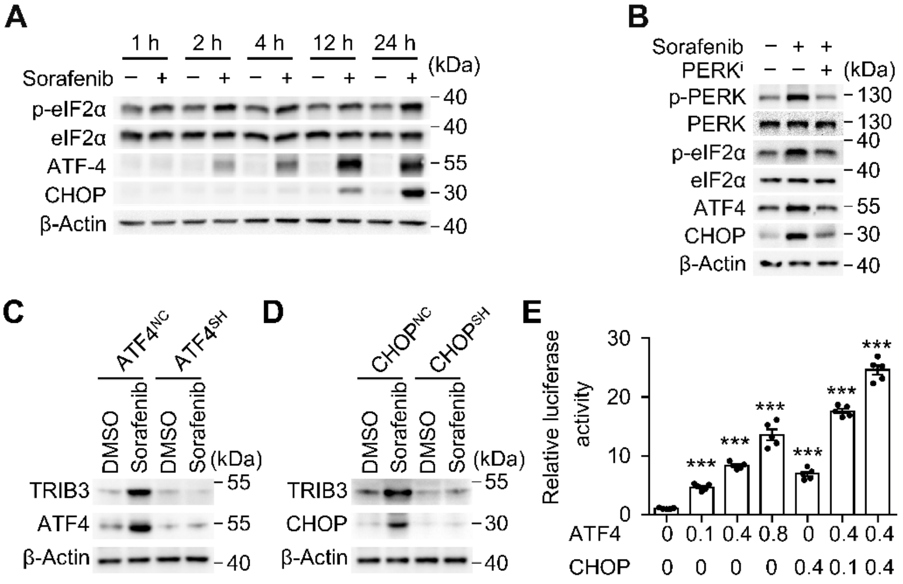

Figure S2. TRIB3 is triggered by sorafenib-elicited ROS-ER stress axis in HCC. A) Huh-7 cells were treated with DMSO or sorafenib for indicated time. Activation of indicated pathways was measured by immunoblotting (*n* = 3). **B**) Huh-7 cells were treated with DMSO or sorafenib in the absence or presence of PERK inhibitor, activation of PERK (1 h) and downstream signaling pathways (48 h) was determined (*n* = 5). **C**, **D**) Wild-type ($ATF4^{NC}$ or $CHOP^{NC}$), or ATF4-knockdown ($ATF4^{SH}$), or CHOP-knockdown ($CHOP^{SH}$) huh-7 cell were treated with DMSO or sorafenib. After 48 h, the cell lysates were analyzed by immunoblotting using anti-TRIB3, anti-ATF4, anti-CHOP and anti-β-actin (*n* = 3). **E**) 293T cells were transiently transfected with pTRIB3-Luc and pRL-TK in combination with the indicated expression vectors for 48 h. Luciferase activity in cell lysates was then measured. The ratio of firefly luciferase activity to renilla luciferase activity was calculated to normalize the data (*n* = 5).

Figure S3

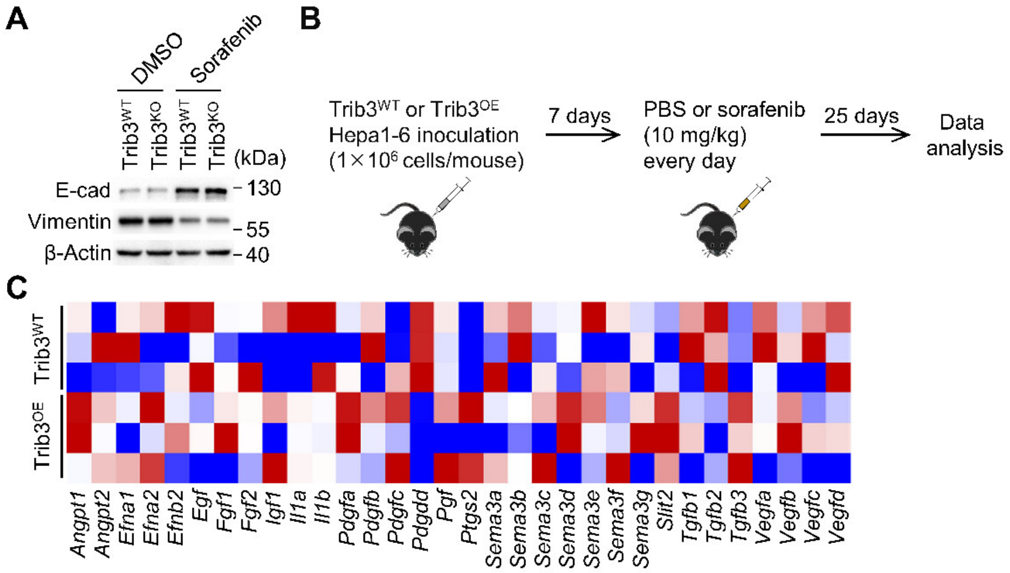

Figure S3. In vivo milieus dictate TRIB3's protumorigenic properties. A) Trib3^{WT} and Trib3^{KO} Hepa1-6 cells were treated with DMSO or sorafenib. Proteins of EMT genes were determined by immunoblotting (*n* = 3). **B**) Trib3^{WT} or Trib3^{OE} Hepa1-6 hepatoma-bearing mice were injected with PBS or sorafenib as described (each *n* = 5). **C**) Heatmap showing the expression of angiogenesis-associated genes in Trib3^{WT} and Trib3^{OE} Hepa1-6 cells.

Figure S4

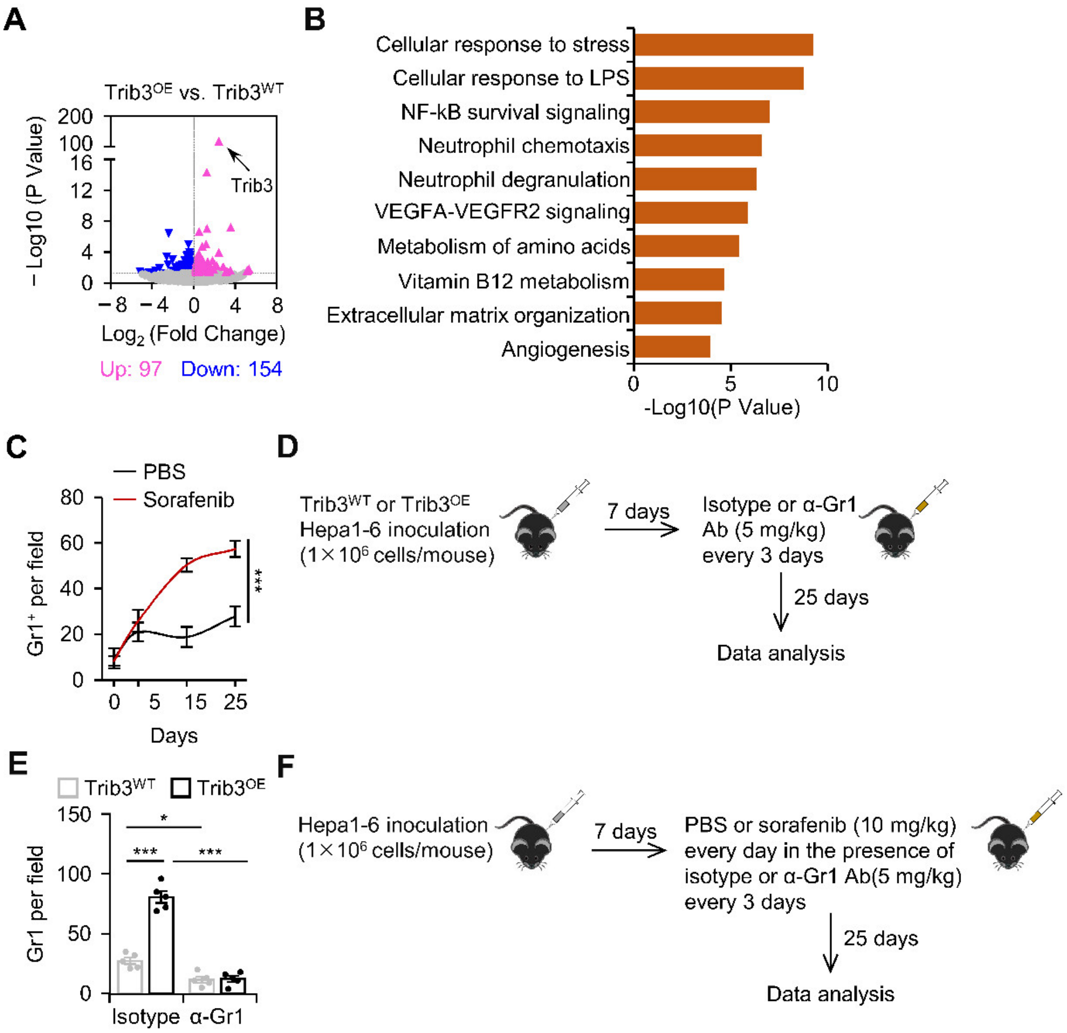

Figure S4. Neutrophils are essential for TRIB3-driven pro-tumorigenesis. A) Volcano plot showing changes in genes expression by in Trib3^{OE} versus Trib3^{WT} hepatoma tissue. The P value of each gene was calculated using DESeq2 and adjusted with Benjamini-Hochberg false discovery rate (FDR) correction. **B**) Functional annotation of 97 upregulated genes expression listed in (**A**) were analyzed by Metascape. The top 10 enrichment terms are listed. **C**) Hepa1-6 hepatoma-bearing mice were injected with PBS or sorafenib. The infiltration of Gr1^{+} neutrophils in the tumor tissues was dynamically assessed by immunohistochemistry (n = 5). **D**, **E**) Trib3^{WT} or Trib3^{OE} Hepa1-6 hepatoma-bearing mice were treated with αGr-1 or isotype antibody (Ab) as described (**D**). Gr-1^{+} neutrophil infiltration in tumor were analyzed (**E**, n = 5). **F**) Hepa1-6 hepatoma-bearing mice were injected with sorafenib or PBS in the presence or absence of αGr-1 Ab as described.

Data represent mean ± SEM of three independent experiments (**C**, **E**). *p < 0.05, ***p< 0.001, two-way ANOVA with Tukey's post test (**E**).

Figure S5

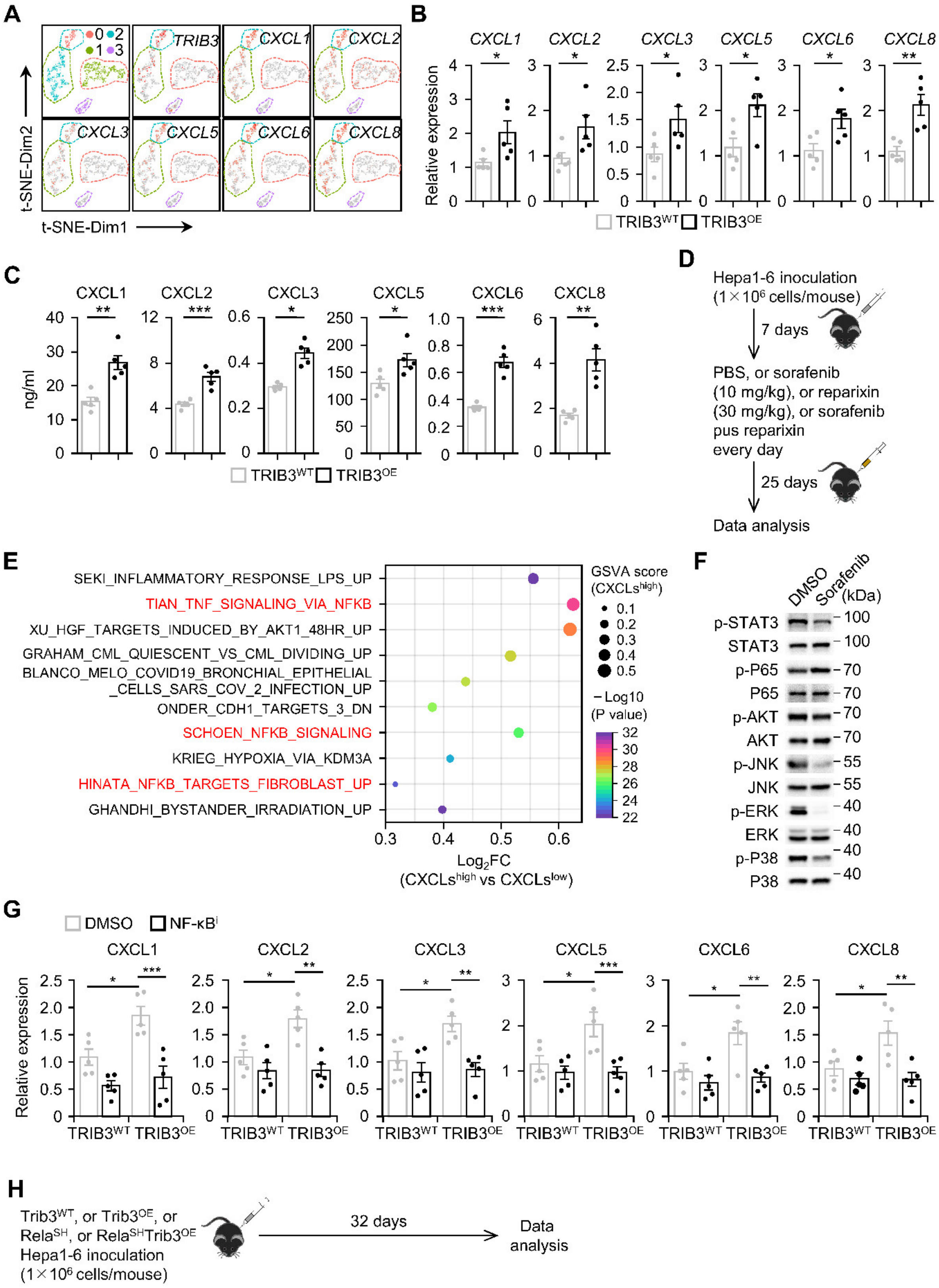

A
B
C
D
E
F
G
H
Hepa1-6 inoculation
(1×10^6 cells/mouse)
7 days
PBS, or sorafenib
(10 mg/kg), or reparixin
(30 mg/kg), or sorafenib
pus reparixin
every day
25 days
Data analysis
TRIB3WT TRIB3OE
DMSO NF-κBi
Trib3WT, or Trib3OE, or
RelaSH, or RelaSHTrib3OE
Hepa1-6 inoculation
(1×10^6 cells/mouse)
32 days
Data
analysis

Figure S5. Elevated TRIB3 attracts neutrophils through boosting NF-κB-CXCL signaling. A) t-Distributed Stochastic Neighbor Embedding (t-SNE) of HCC malignant cells, with each cell colored based on the clustering or expression of *TRIB3*, *CXCL1*, *CXCL2*, *CXCL3*, *CXCL5*, *CXCL6*, *CXCL8*. (GEO: GSE125449). **B**) Effects of TRIB3-overexpression on expression of CXCR1/2 ligands in Huh-7 cells were analyzed by RT-PCR (**B**) and ELISA (**C**) (n = 5). **D**) Hepa1-6 hepatoma-bearing mice were injected with PBS or sorafenib in the absence or presence of CXCR1/2 inhibitor (Reparixin) as described (each n = 5). **E**) 366 HCC samples from TCGA dataset were divided into two groups according to the expression of CXCL1, CXCL2, CXCL3, CXCL5, CXCL6, CXCL8: CXCLs^{high} (samples with overall higher expression of CXCL1/2/3/5/6/8 than the median of 366 samples, n = 61) and CXCLs^{low} (samples with overall lower expression of CXCL1/2/3/5/6/8 than the median of the 366 samples, n = 76). Pathway activities in the 143 patients were calculated by Gene Set Variation Analysis (GSVA) of a total of 7233 C2 curated genesets obtained from the Human Molecular Signatures Database (MSigDB).Top 10 enriched pathways in CXCLs^{high} patients were listed. **F**) Huh-7 cells were treated with DMSO or sorafenib for 48 hours. Activation of indicated pathways was analyzed by immunoblotting (n = 4). **G**) Expression of CXCR1/2 ligands in wild-type Huh-7 cells (TRIB3^{WT}) or TRIB3-overexpressing Huh-7 cells (TRIB3^{OE}) treated with DMSO or sorafenib in the presence of DMSO or NF-κB inhibitor (JSH-23, 50 μm) for 48 h (n = 5). **H**) Wild-type (Trib3^{WT} or Rela^{NC}), or Trib3-overexpressing (Trib3^{OE}), or p65-knockdown (Rela^{SH}), or p65-knockdown plus Trib3-overexpressing ($\text{Rela}^{SH}\text{Trib3}^{OE}$) Hepa1-6 cells were inoculated in liver of C57BL/6 mice as described (each n = 5). Data represent mean ± SEM of three independent experiments (**B, C, G**). *p <0.05, **p <0.01, **p <0.001, Student's t test (**B, C**), two-way ANOVA with Tukey's post test (**G**).

Figure S6

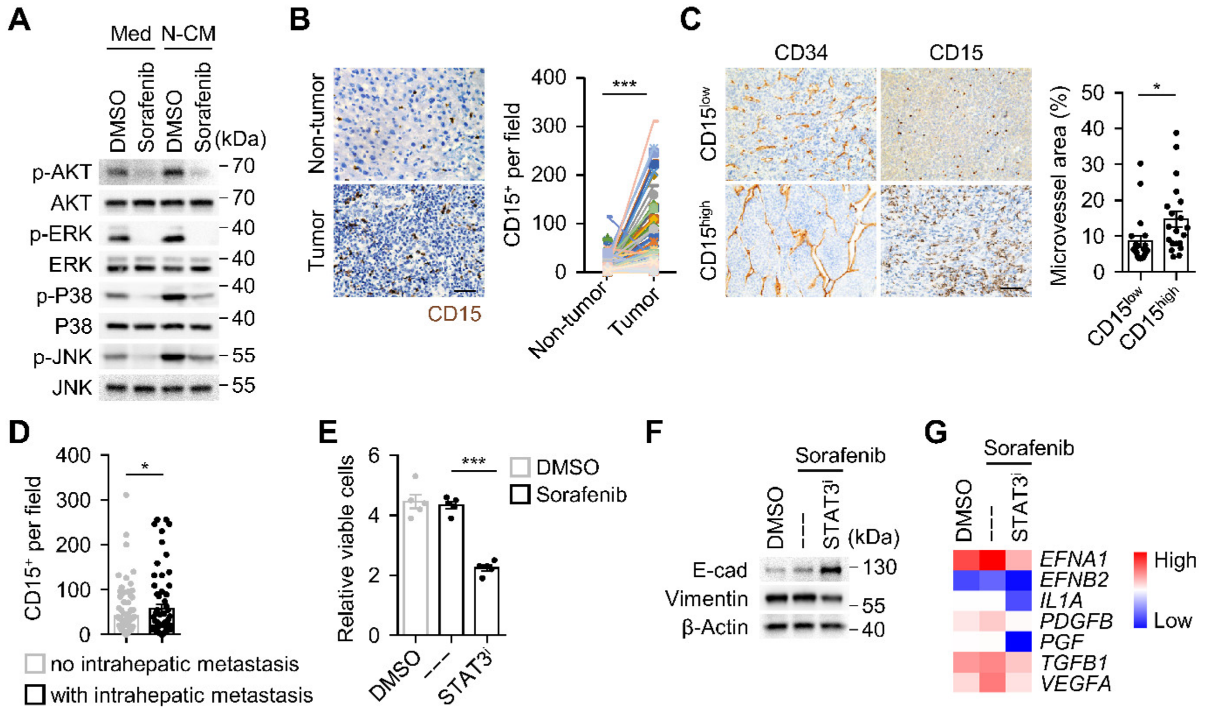

Figure S6. Neutrophils suppress therapeutic efficacy of sorafenib via OSM/STAT3 axis. A) Huh-7 cells were treated with DMSO or sorafenib in the absence or presence of conditioned medium from tumor-associated neutrophils (N-CM). Activation of indicated pathways was determined by immunoblotting (*n* = 3). **B**) Distribution of CD15$^+$ cells in nontumoral liver and tumor tissue of HCC samples (*n* = 148). Scale bar, 50 µm. **C**) Association of infiltration levels of CD15$^+$ neutrophils with angiogenesis in HCC tumors (*n* = 40). Scale bar,100 µm. **D**) Association of infiltration levels of CD15$^+$ neutrophils in HCC tumors with intrahepatic metastasis (no intrahepatic metastasis, *n* = 80; with intrahepatic metastasis, *n* = 35). **E–G**) Huh-7 cells were treated with DMSO, or sorafenib, or sorafenib plus STAT3 inhibitor (STAT3^i: AG490, 100 µm) in the presence of conditioned medium from tumor-associated neutrophils (N-CM) (*n* = 5). Cell proliferation (**E**), proteins of EMT genes (**F**), and expression of angiogenesis-related genes (**G**) were determined. Data represent mean ± SEM of three independent experiments (**C–E**). *p <0.05, ***p <0.001, Student's t test (**B-D**), one-way ANOVA with Tukey's post test (**E**).

Supplementary Tables

Table S1. Clinical characteristics of 196 hepatocellular carcinoma patients

Patient characteristics	Cohort 1	Cohort 2	Cohort 3
No. of patients	152	14	30
Age, y, median (range)	53 (19 - 81)	52 (27 -70)	48 (30 - 78)
Sex, male / female, n	131 / 21	12 / 2	25 / 5
HBsAg, negative / positive, n	29 / 123	5 / 9	6 / 24
Cirrhosis, absent / present, n	31 / 121	7 / 7	7 / 23
ALT, U/L, median (range)	40.3 (13.9 - 172.8)	30.7 (9.6 - 69.4)	41 (1.3 - 129.3)
AFP (≤ 25 ng/mL / > 25 ng/mL), n	68 / 84	6 / 8	16 / 14
Tumor size (≤ 5 cm / > 5 cm), n	66 / 86	7 / 7	7 / 23
Tumor multiplicity, solitary / multiple, n	125 / 27	11 / 3	21 / 9
Vascular invasion, absent / present, n	106 / 46	8 / 6	8 / 22
TNM stage, I + II / III + IV, n	125 / 27	11 / 3	20 / 10
Tumor differentiation, I + II / III + IV, n	69 / 83	7 / 7	12 / 18
Fibrous capsule, absent / present, n	51 / 101	5 / 9	7 / 23

Abbreviations: HBsAg, hepatitis B surface antigen; AFP, alpha fetoprotein; ALT, alanine aminotransferase; TNM, tumor-node-metastasis.

Note: Samples from patients in Cohort 1 were used in Figure 3H, Figure 4B, Figure S6B-D; Samples from patients in Cohort 2 were used in Figure 6A–D, Figure 6F,G, Figure S6A, Figure S6E–G; Samples from patients in Cohort 3 were used in Figure S1F,G.

Table S2. Characteristics of 14 patients contributing fresh samples

No	Age	Sex	HBsAg	HBsAb	HBeAg	HBeAb	HBcAb	HBV DNA (copy/ml)	HCVAb	Liver Cirrhosis	TNM Stage	Histological Grade	AFP (ng/ml)
1	61	M	+	+	-	+	+	0	-	-	T1N0M0	I	2.14
2	60	M	-	+	-	-	-	0	-	-	T1N0M0	II	376.4
3	70	M	-	+	-	+	+	0	-	+	T1N0M0	II	3.73
4	55	M	+	-	-	+	+	1020	-	+	T1N0M0	III	5.29
5	52	M	+	-	-	-	-	980000	-	+	T2N0M0	II	7.44
6	43	M	+	-	-	-	-	75.20	-	+	T1N0M0	III	172
7	47	M	+	-	-	-	+	179	-	+	T1N0M0	III	6362
8	47	M	-	-	-	-	-	0	-	+	T2N0M0	III	2.44
9	52	M	+	-	-	-	-	0	-	-	T2N0M0	II	2132
10	55	F	+	-	-	-	+	5390	-	-	T3N0M0	II	85.06
11	69	F	-	+	-	+	+	0	-	-	T3N0M0	II	9422
12	41	M	+	-	-	+	+	919	-	-	T1N0M0	III	316.3
13	27	M	+	-	+	-	-	554	-	+	T3N0M0	III	100.7
14	55	M	-	-	-	-	-	0	-	-	T2N0M0	IV	1.81

Table S3. Characteristics of 30 patients contributing twice samples in Figure 1B,C

No	Sex	History of Hepatitis B	Primary sample collection						Days to primary sample collection	Sorafenib therapy prior to secondary resection	Lung metastasis before secondary resection
			Age	Liver Cirrhosis	Lung metastasis	Tumor multiplicity	TNM Stage	Histological Grade			
1	M	YES	46	Absent	NO	Solitary	T2N0M0	III	737	NO	NO
2	M	YES	63	Present	NO	Solitary	T1N0M0	III	139	NO	NO
3	M	YES	53	Present	NO	Solitary	T2N0M0	II	154	NO	NO
4	M	YES	66	Present	NO	Solitary	T1N0M0	III	175	NO	NO
5	M	YES	36	Present	NO	Multiple	T3N0M0	II	136	NO	NO
6	F	YES	44	Absent	NO	Solitary	T2N0M0	III	537	NO	NO
7	M	YES	73	Present	NO	Solitary	T2N0M0	II	118	NO	NO
8	M	YES	51	Absent	NO	Multiple	T3N0M0	II	269	NO	YES
9	M	NO	54	Present	NO	Solitary	T1N0M0	III	124	NO	NO
10	M	YES	50	Present	NO	Solitary	T1N0M0	III	268	NO	NO
11	M	YES	54	Present	NO	Solitary	T2N0M0	II	348	NO	NO
12	M	NO	46	Present	NO	Solitary	T1N0M0	II	110	NO	NO
13	M	YES	49	Present	NO	Solitary	T1N0M0	II	378	NO	NO
14	M	YES	61	Present	NO	Solitary	T1N0M0	II	160	NO	NO
15	M	YES	72	Present	NO	Solitary	T2N0M0	III	325	NO	NO
16	M	YES	55	Absent	NO	Multiple	T3N0M0	III	168	YES	NO
17	M	YES	53	Absent	NO	Solitary	T2N0M0	II	625	YES	NO
18	M	YES	58	Absent	YES	Solitary	T4N0M0	III	192	YES	YES
19	M	YES	25	Present	NO	Solitary	T2N0M0	III	378	YES	YES
20	M	YES	48	Present	NO	Solitary	T2N0M0	III	748	YES	NO
21	M	YES	51	Present	NO	Solitary	T2N0M0	III	607	YES	NO
22	M	YES	39	Present	NO	Solitary	T2N0M0	III	138	YES	NO
23	M	YES	55	Absent	NO	Solitary	T2N0M0	III	939	YES	NO
24	M	YES	34	Absent	NO	Multiple	T3N0M0	III	765	YES	NO
25	M	YES	65	Present	NO	Solitary	T2N0M0	III	492	YES	NO

No	Sex	History of Hepatitis B	Primary sample collection						Days to primary sample collection	Sorafenib therapy prior to secondary resection	Lung metastasis after sorafenib
			Age	Liver Cirrhosis	Lung metast asis	Surgical Margin Resection Status	TNM Stage	Histological Grade			
26	M	YES	43	Absent	NO	Solitary	T2N0M0	III	470	YES	NO
27	M	YES	25	Present	NO	Solitary	T4N0M0	III	346	YES	YES
28	M	YES	42	Absent	NO	Multiple	T3N0M0	II	169	YES	YES
29	M	YES	61	Absent	NO	Multiple	T4N0M0	II	999	YES	NO
30	M	YES	43	Present	NO	Solitary	T2N0M0	II	161	YES	NO

Table S4. Antibodies for immunohistochemistry, immunofluorescence and flow cytometry

Antibody	Supplier	Identifier
Anti-Human TRIB3 Antibody	Abcam	Cat# ab137526, RRID: AB_2876352
Anti-Human CD15 Antibody	ZSBio	Cat# ZM-0037, RRID: AB_2924229
Anti-Human CD34 Antibody	ZSBio	Cat# ZM-0046, RRID: AB_2924244
Anti-Mouse Ki67 Antibody	Abcam	Cat# ab16667, RRID: AB_302459
Anti-Mouse CD31 Antibody	Cell Signaling Technology	Cat# 77699, RRID: AB_2722705
Anti-Mouse B220 Antibody (Clone RA3-6B2)	BD Biosciences	Cat# 557390, RRID:AB_396673
Anti-Mouse CD4 Antibody (Clone EPR19514)	Abcam	Cat# ab183685, RRID:AB_2686917
Anti-Mouse CD8 Antibody (Clone D4W2Z)	Cell Signaling Technology	Cat# 98941, RRID:AB_2756376
Anti-Mouse F4/80 Antibody (Clone BM8)	Thermo Fisher Scientific	Cat# MF48000, RRID:AB_10376289
Anti-Mouse Ly-6G/Ly-6C Antibody (Clone RB6-8C5)	Thermo Fisher Scientific	Cat# 14-5931-85, RRID:AB_467731
Anti-Mouse NKp46 Antibody (Clone EPR23097-35)	Abcam	Cat# ab233558, RRID:AB_2904203
Anti-Human CD14 Antibody, AF 700, Clone M5E2	BD Biosciences	Cat# 557923, RRID:AB_396944
Anti-Human CD19 Antibody, BV421	BioLegend	Cat# 302234, RRID:AB_11142678
Anti-Human CD3 Antibody, AF 700, Clone OKT3	eBioscience	Cat# 56-0032-82, RRID:AB_529507
Anti-Human CD45 Antibody, KO, Clone J.33	Beckman Coulter	Cat# A96416, RRID:AB_2888654
Anti-Human CD56 Antibody, PC7, Clone N901	Beckman Coulter	Cat# A21692, RRID:AB_2892144

Table S5. Antibodies for immunoblotting

Antibody	Supplier	Identifier
Anti-β-Actin Antibody	Origene	Cat# TA310155, RRID: AB_10691552
Anti-TRIB3 Antibody	Thermo Fisher Scientific	Cat# PA5-114501 RRID:AB_2890514
Anti-DDDDK-tag Antibody	MBL International	Cat# M185-3L, RRID:AB_11123930
Anti-PERK Antibody	Cell Signaling Technology	Cat# 3192, RRID:AB_2095847
Anti-phospho-PERK Antibody	Affinity Biosciences	Cat# DF7578, RRID:AB_2841070
Anti-ATF4 Antibody	Cell Signaling Technology	Cat# 11815, RRID:AB_2616025
Anti-ATF6 Antibody	Cell Signaling Technology	Cat# 65880, RRID:AB_2799696
Anti-IRE1α Antibody	Cell Signaling Technology	Cat# 3294, RRID:AB_823545
Anti-phospho-IRE1 Antibody	Abcam	Cat# ab48187, RRID:AB_873899
Anti-phospho-eIF2α Antibody	Cell Signaling Technology	Cat# 9721, RRID:AB_330951
Anti-eIF2α Antibody	Cell Signaling Technology	Cat# 2103, RRID:AB_836874
Anti-CHOP Antibody	Cell Signaling Technology	Cat# 2895, RRID:AB_2089254
Anti-phospho-Akt Antibody	Cell Signaling Technology	Cat# 4060, RRID: AB_2315049
Anti-Akt Antibody	Cell Signaling Technology	Cat# 4691, RRID: AB_915783
Anti-E-Cadherin Antibody	BD Biosciences	Cat# 610181, RRID:AB_397580
Anti-phospho-Erk1/2 Antibody	Cell Signaling Technology	Cat# 4370, RRID: AB_2315112
Anti-Erk1/2 Antibody	Cell Signaling Technology	Cat# 4695, RRID: AB_390779
Anti-phospho-JNK Antibody	Cell Signaling Technology	Cat# 4668, RRID: AB_823588
Anti-JNK Antibody	Cell Signaling Technology	Cat# 9252, RRID: AB_2250373
Anti-phospho-p38 Antibody	Cell Signaling Technology	Cat# 4511, RRID: AB_2139682
Anti-p38 Antibody	Cell Signaling Technology	Cat# 8690, RRID: AB_10999090
Anti-phospho-P65 Antibody	Cell Signaling Technology	Cat# 3033, RRID: AB_331284
Anti-P65 Antibody	Cell Signaling Technology	Cat# 8242, RRID: AB_10859369
Anti-Stat3 Antibody	Cell Signaling Technology	Cat# 9139, RRID: AB_331757
Anti-phospho-Stat3 Antibody	Cell Signaling Technology	Cat# 9145, RRID: AB_2491009
Anti-Vimentin Antibody	BD Biosciences	Cat# 550513, RRID: AB_393716

Table S6. Recombinant proteins, peptides, chemicals, and critical commercial assays

Name	Supplier	Identifier
**Chemicals**		
Sorafenib	Selleck	Cat# S7397
Reparixin	Selleck	Cat# S8640
AG490	Selleck	Cat# S1143
GSK2606414	Selleck	Cat# S7307
JSH-23	Sigma	Cat# J4455
N-Acetyl-L-cysteine	Sigma-Aldrich	Cat# A9165
**Recombinant proteins, peptides**		
InVivoMAb anti-mouse Ly6G	Bio X Cell	Cat# BE0075-1
Goat IgG	R and D Systems	Cat# AB-108-C
Mouse IgG	R and D Systems	Cat# 20102
Human OSM Neutralizing Antibody	R and D Systems	Cat# MAB295-500
Mouse OSM Neutralizing Antibody	R and D Systems	Cat# AF-495-NA
**Critical commercial assays**		
CD15 MicroBeads	Miltenyi	Cat# 130-046-601
5X All-In-One RT MasterMix	Abm	Cat# G492
Hieff qPCR SYBR Green Master Mix	Yeasen Biotech	Cat# 11201ES03
D-Luciferin	Goldbio	Cat# LUCK-1G
CCK8	Beyotime	Cat# C0039
DCFH-DA	Sigma	Cat# D6883
AnnexinV	BioVision	Cat# 1001-1000
Propidium Iodide	Invitrogen	Cat# P1304MP
Dual Luciferase Reporter Gene Assay Kit	Yeasen	Cat# 11402ES60
Human CXCL1 ELISA kit	Genkern	Cat# GK-E0128H
Human CXCL2 ELISA kit	Genkern	Cat# GK-E0188H
Human CXCL3 ELISA kit	Genkern	Cat# GK-E0619H
Human CXCL5 ELISA kit	Genkern	Cat# GK-E0130H
Human CXCL6 ELISA kit	Genkern	Cat# GK-E0446H
Human CXCL8 ELISA kit	Genkern	Cat# GK-E0008H
Human OSM ELISA kit	R and D Systems	Cat# DY295

Table S7. Sequence-based reagents

Gene	Forward	Reverse
**RT-PCR**		
human-*CXCL1*	CTTGCCTCAATCCTGCATC	CCTTCTGGTCAGTTGGATTTG
human-*CXCL2*	GCCCAAACCGAAGTCATAG	CAGGAACAGCCACCAATAAG
human-*CXCL3*	CACTCAAGAATGGGAAGAAAG	CTGCAGGAAGTGTCAATGATAC
human-*CXCL5*	TGGTAGCCTCCCTGAAGAAC	AGACTGGGAAACTTTTCCATG
human-*CXCL6*	AGAGCTGCGTTGCACTTGTT	GCAGTTTACCAATCGTTTTGGGG
human-*CXCL8*	ACTGAGAGTGATTGAGAGTGGA	AACCCTCTGCACCCAGTTTTC
human-*EFNA1*	TCAGGCCCATGACAATCCAC	GTGACCGATGCTATGTAGAACC
human-*EFNB2*	TTCAGCCCTAACCTCTGGGG	CCTCCAAAGACCCATTTGATGTA
human-*IL1A*	AGATGCCTGAGATACCCAAAAC	CCAAGCACACCCAGTAGTCT
human-*PDGFB*	CTCGATCCGCTCCTTTGATGA	CGTTGGTGCGGTCTATGAG
human-*PGF*	GAACGGCTCGTCAGAGGTG	ACAGTGCAGATTCTCATCGCC
human-*TGFB1*	CTAATGGTGGAAACCCACAACG	TATCGCCAGGAATTGTTGCTG
human-*VEGFA*	AGGGCAGAATCATCACGAAGT	AGGGTCTCGATTGGATGGCA
human-*TRIB3*	TGCCCTACAGGCACTGAGTA	GTCCGAGTGAAAAAGGCGTA
human-*ACTIN*	GGATGCAGAAGGAGATCACT	CGATCCACACGGAGTACTTG
mouse-*TRIB3*	GCCTACGTGGGACCAGAGATAC	CTCCAGACATCAGCCGCTTT
mouse-*ACTIN*	GGATGCAGAAGGAGATCACT	CGATCCACACGGAGTACTTG
**shRNA**		
sh-human-*TRIB3*	CGGTTGGAGTTGGATGACAACTTAG	CTAAGTTGTCATCCAACTCCAACCG
sh-human-ATF4	GAACAGGAGAATGAAAGGAAAC	GTTTCCTTTCATTCTCCTGTTC
sh-human-CHOP	CCACTCCAGATCATTCCTTTAC	GTAAAGGAATGATCTGGAGTGG
sh-mouse-*Rela*	GCGAATCCAGACCAACAATAA	TTATTGTTGGTCTGGATTCGC
**sgRNA**		
sgTrib3-1	TCTTTTTCACGAAGACCCAT	ATGGGTCTTCGTGAAAAAGA
sgTrib3-2	CTCTTTGGCAAGATCCGTAG	CTACGGATCTTGCCAAAGAG
